# From (Sub)Porphyrins to (Sub)Phthalocyanines: Aromaticity Signatures in the UV–Vis Absorption Spectra

**DOI:** 10.1021/acs.inorgchem.4c03139

**Published:** 2024-09-19

**Authors:** Sílvia Escayola, Jorge Labella, Dariusz W. Szczepanik, Albert Poater, Tomas Torres, Miquel Solà, Eduard Matito

**Affiliations:** † Institut de Química Computacional i Catàlisi and Departament de Química, Universitat de Girona, C/Maria Aurèlia Capmany, 69, Girona, Catalonia 17003, Spain; ‡ Donostia International Physics Center (DIPC), Donostia, Euskadi 20018, Spain; § Departamento de Química Orgánica, Universidad Autónoma de Madrid, Madrid 28049, Spain; ∥ Department of Theoretical Chemistry, Faculty of Chemistry, Jagiellonian University, Kraków 30-387, Poland; ⊥ Institute for Advanced Research in Chemical Sciences (IAdChem), Universidad Autónoma de Madrid, Madrid 28049, Spain; # IMDEA-Nanociencia, Campus de Cantoblanco, Madrid 28049, Spain; ∇ Ikerbasque Foundation for Science, Bilbao, Euskadi 48011, Spain

## Abstract

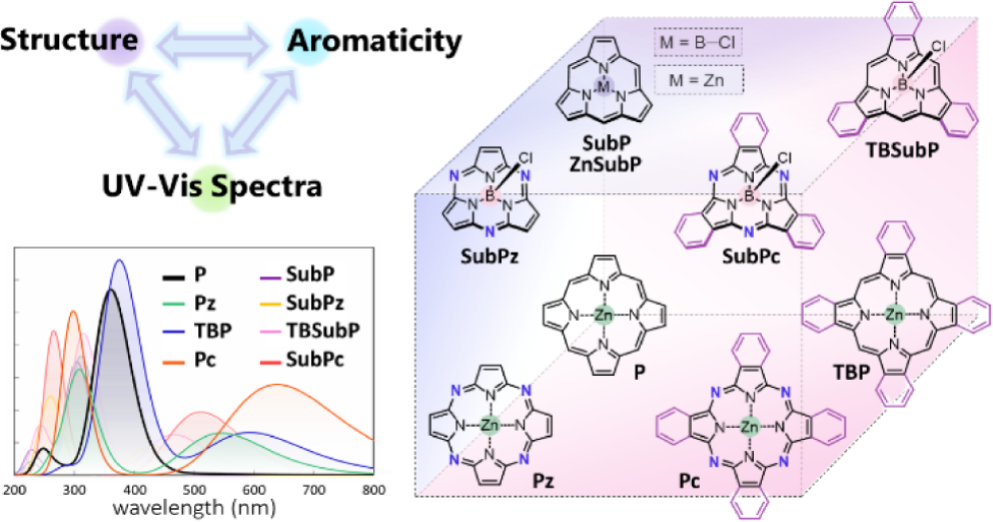

The development of novel synthetic methods has greatly expanded the toolbox available to chemists for engineering porphyrin and phthalocyanine derivatives with precise electronic and optical properties. In this study, we focus on the UV–vis absorption characteristics of substituted phthalocyanines and their contracted analogs, subphthalocyanines, which feature nonplanar, bowl-shaped geometries. These macrocycles, which are central to numerous applications in materials science and catalysis, possess extensive π-conjugated systems that drive their unique electronic properties. We explore how the change from a metalloid (B) to a metal (Zn) and the resulting coordination environments influence the aromaticity and, consequently, the spectroscopic features of these systems. A combined computational and experimental approach reveals a direct correlation between the aromaticity of the external conjugated pathways and the Q bands in the UV–vis spectra. Our findings highlight key structural modifications that can be leveraged to fine-tune the optical properties of porphyrinoid systems, offering new pathways for the design of advanced materials and catalysts with tailored functionalities.

## Abstract 2

Exploring structural variations in zinc and boron-hydride coordinated (sub)porphyrin and (sub)phthalocyanine systems while uncovering correlations between spectroscopic features and aromatic pathways.

## Introduction

**Scheme 1:**
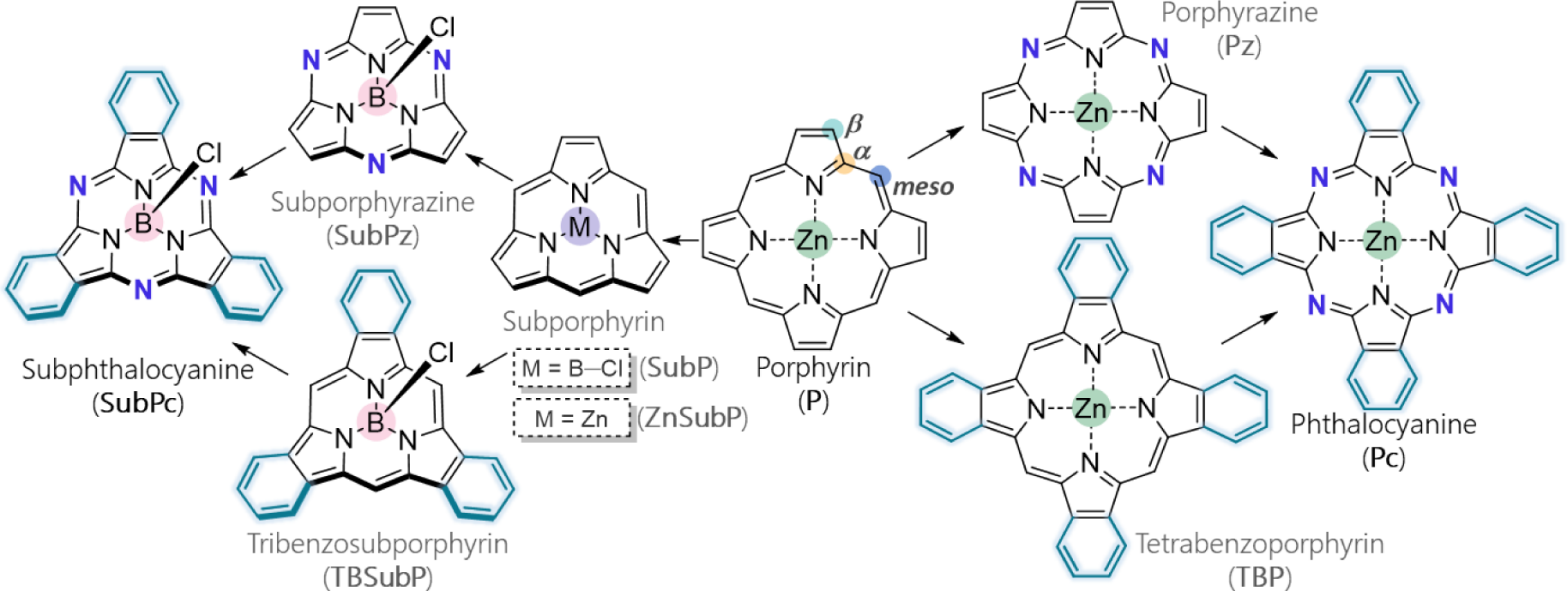
Metallo or B–X Coordinated (Sub)Porphyrins and (Sub)Phthalocyanines Included in This Study The structural differences that relate porphyrin with phthalocyanine and subphthalocyanine are highlighted in dark blue (*N–meso*) and turquoise (fused 6-MR). In subporphyrin, we considered both central Zn and B–Cl coordination.

Over the last century, the continuous improvement of synthetic methodologies for generating porphyrin derivatives converged to the point where these can be obtained based on targeted properties.^1^ Among the wide variety of systems, some interesting examples are phthalocyanines (Pcs),^2−4^ porphyrazines (Pzs),^5,6^ tetrabenzoporphyrins (TBPs),^7^ and their respective ring-contracted versions, subporphyrins (SubPs),^8^ subphthalocyanines (SubPcs),^8−10^ subporphyrazines (SubPzs),^9^ and tribenzosubporphyrins (TBSubPs),^11^ see Scheme 1. The former are aromatic and composed of four isoindole units, interconnected via nitrogen (N) or methine (=CH−) bridges (at the *meso* positions, Scheme 1)^12,13^ and tend to be highly planar unless distortion is forced by the addition of bulky substituents or large metal ions, as observed in some metal-substituted Pcs and TBPs.^14,15^ The latter, also aromatic, only have three isoindole moieties and adopt nonplanar bowl-shaped geometries.^16^

**Figure 1:**
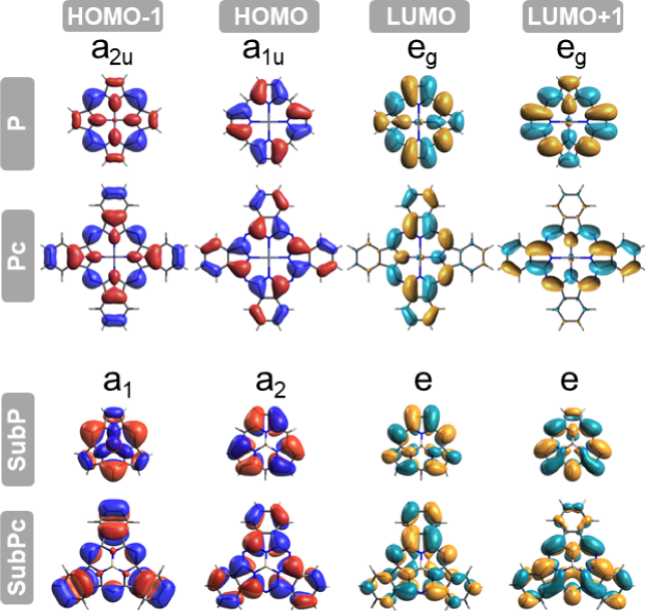
Spatial representation of frontier a_2u_, a_1u_, and e_g_ (or a_1_, a_2_, and e in C_3*v*_) molecular orbitals, with an isocontour of 0.02 a.u., from top to bottom for **P**, **Pc**, **SubP**, and **SubPc**. In the case of **SubPc**, the a_1_ orbital corresponds to the HOMO – 3.

Key features of these macrocyclic compounds are their extended π-conjugated system and central coordination, which are responsible for their unique properties. Compared to porphyrin, (Sub)Pcs exhibit characteristic ultraviolet–visible (UV–vis) absorption spectra, with (blue)red-shifted Q bands and blue-shifted Soret, or B, bands. According to the Gouterman four-orbital model for porphyrins,^17−19^ Q and B bands arise from π–π* transitions and can be understood by considering the four frontier orbitals: a_2u_, a_1u_, and two e_g_ (corresponding to HOMO – 1, HOMO, LUMO, and LUMO + 1, which will be referred to as H – 1, H, L, and L + 1, respectively), depicted in Figure 1. The different orbital mixing splits the resulting excited states into lower-energy, Q bands (S_0_ → S_1_), and higher-energy, Soret bands (S_0_ → S_2_).^20^ The central metal coordination affects these spectra by altering the overlap between the metal and ligand orbitals, leading to variations in their energy gaps and thus influencing the position of the absorption bands.^21^ For instance, nickel porphyrins have similar ring currents to their zinc analogs, but due to their vacant d_*x*_^2^_–y_^2^ orbitals they tend to have larger HOMO–LUMO gaps and lower HOMO levels, resulting in blue-shifted absorption spectra and lower chemical reactivity.^22^

The typical absorption spectra of metalloporphyrins consist of two weak Q bands at 500–650 nm and a Soret intense band at 370–450 nm.^20^ In the case of metallophthalocyanines, Q and B bands lie around 600–800 and 300–400 nm, respectively, whereas in subphthalocyanines, Q and B bands appear at 460–560 and 260–370 nm regions.^9^ In Zn-phthalocyanine (**Pc**) and subphthalocyanine (**SubPc**), the relative intensity of Q and Soret bands is reversed compared to Zn-porphyrin (**P**). This change has been primarily attributed to the N–*meso* substitution that breaks the a_2u_–a_1u_ near-degeneracy, selectively stabilizing the a_2u_ orbital, thereby increasing the intensity of the Q-band.^23−25^ Their characteristic UV–vis spectra, low-lying singlet (S_1_)–triplet (T_1_) energy gaps (), H–L gaps, and other properties (e.g., conductance)^26^ make them optimal candidates for solar cells,^3,27−30^ nonlinear optics,^31^ molecular electronics,^32^ and photonics.^33,34^ Furthermore, (sub)phthalocyanine derivatives are unique photoactive materials to prepare energy and electron donor–acceptor systems.^35^ In this regard, notable is the use of **Pcs** complexing heavy metals for triplet–triplet annihilation upconversion (TTA-UC),^36−39^ and the use of **SubPcs** and **SubPzs** for singlet-fission downconversion (SF-DC).^40−42^ Overall, these compounds are promising materials for a wide array of applications. However, a clear establishment of structure–property–and property–property relationships is crucial to fully exploiting their highly tunable potential and applications. Some examples along the lines of identifying these relationships are the independent studies of Zhang et al.,^23^ Belosludov et al.,^43^ Holst et al.,^44^ and Peterson et al.,^45^ where they found a correspondence between computed H–L gaps and spectroscopic properties or variations in S_1_ and T_1_ state energies. The mere calculation of H–L gaps might not be adequate to pinpoint these properties due to potential accuracy issues, as highlighted by Holst and co-workers.^44^ There is an ongoing need for streamlined methods that enable faster screening through alternative computational analyses. Several authors have unveiled connections between the (anti)aromaticity in diverse free-base or metal substituted porphyrinoids and their UV–vis or infrared spectra,^46^ with some focusing on nonlinear optical properties.^47−53^ Ke et al. recently introduced a way to regulate the properties of silicon(IV) phthalocyanines by switching their aromaticity.^54^ These discoveries hint at a potential interplay between the aromaticity and properties of porphyrinoids. Expanding upon this research, we propose the use of chemical bonding and aromaticity analyses as a systematic procedure to identify π-system–UV–vis absorption and  correlations in porphyrin-related compounds, improving the understanding of physical properties and reactivity in these complexes.

Aromaticity is widely acknowledged as a pivotal concept in characterizing electronic structures,^55−59^ the Hückel rule (4*N* + 2)^60−62^ offering the most straightforward approach to predicting the aromatic nature of molecules. While the application of the Hückel rule is primarily focused on planar monocyclic molecules, such as annulenes and their analogues,^63,64^ its simplicity spurred researchers to modify it for intricate systems.^65−68^ Traditionally, the aromaticity of porphyrins and **Pcs** has been ascribed to an 18π-electron aromatic cycle (and a 14π-electron cycle in **SubPs** and **SubPcs**) akin to [18]annulene, adhering to the Hückel rule.^69,70^ Obviously, this rule cannot differentiate among molecules with an identical number of π-electrons, and falls short when accounting for the aromaticity of some nonplanar systems; other tools are becoming essential to comprehensively address aromaticity.^47,48,50,51,71−73^ Aromaticity investigations of **Pcs** and, especially, **SubPcs** are sparse and primarily restricted to nucleus-independent chemical shift (NICS) and the harmonic oscillator model of aromaticity (HOMA).^74−78^ Given the intricacy of these molecules—attributable to their size, topology, and the presence of multiple π-electron circuits—and the inherent limitations of NICS and HOMA as aromaticity gauges,^79−81^ there is a compelling case for using more reliable aromaticity descriptors. A more holistic method, integrating both global and local aromaticity metrics, remains desirable to unveil the most favorable pathways for electron delocalization in **Pcs** and **SubPcs**.

**Scheme 2:**
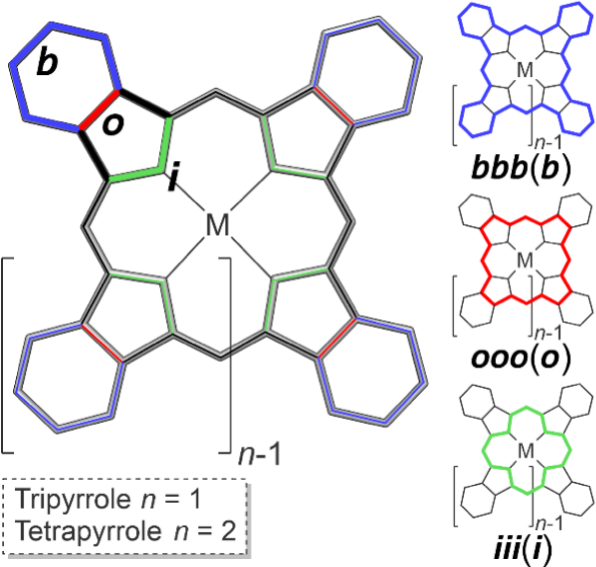
Possible Routes to Follow, i *Inner*, o *Outer*, and b *Benzo*, at Each Pyrrole or Isoindole Moiety, Which Define the Closed Pathways along the Molecule Three examples are the bbb(b), ooo(o), and iii(i) pathways in blue, red, and green, respectively.

One of the main challenges in the description of aromaticity in porphyrinoids is the identification of the most conjugated pathway among the complex ring constructed of bridged rings (including but not limited to pyrrole, isoindole, and derivatives), a task that is not suited for some popular aromaticity indicators such as global NICS analysis. The molecule can be divided into different regions, including *benzo* (b), *outer* (o), and *inner* (i), as defined in Scheme 2. From these regions, potential circuits emerge. Determining the key pathways in such a complex system requires careful analysis and consideration of all possible routes. Over the past few years, significant efforts have been dedicated to the development and application of specific electronic indices to large rings.^71−73,82,83^ The latter need emerges from the inadequacy of the most reliable indices of aromaticity^79^ for their application to ring structures with more than 14 atoms.^82^

In this work, we employ aromaticity indices to identify the key conjugated pathways and discuss the similarities and differences between **P** and **Pc** or **SubPc**. Considering the intrinsic connection between UV–vis absorption spectrum and H–L gap in these molecules and the relationship this gap maintains with the aromaticity of π-conjugated systems,^84,85^ we will investigate a previously unexplored correlation between UV–vis absorbance and local pathways, which holds the promise to serve as a powerful tool for property-guided molecular design. Additionally, we also study the relationship between aromaticity in the singlet ground state and the excited-singlet–triplet gap, . The latter is particularly pertinent given the rising utility of this compound family in applications like triplet photosensitizers,^86^ optoelectronic components,^77^ and photodynamic therapy.^87,88^ Our final goal is to establish the connection between aromaticity and UV–vis absorption spectra or , and identify the molecular segments that are key for the control of electron delocalization, offering a promising avenue to suggest specific modifications, leveraging cost-efficient DFT over TDDFT or more accurate wave function methods that require a full study of the excited states.

## Results

In the following, we present the results of our investigation, comparing the structural differences among **P**, **Pc**, and **SubPc**. These differences (shown in Scheme 1) include (*i*) the replacement of CH at the *meso* position by N, (*ii*) the inclusion of C_4_H_4_ fragments at the β positions to have isoindoles instead of pyrroles, (*iii*) the reduction in the number of pyrrole or isoindole units from four to three, and (*iv*) the replacement of the central Zn atom by the B–Cl moiety in **SubPc** compared to **P** and **Pc**. This study does not chiefly address the role of the central atom and its axial ligation or the effect of peripheral substitutions, which are other common structural changes that tune these molecules. While these modifications can also affect the molecular properties, their impact on the π-system is typically less pronounced compared with the modifications considered. We focused on Zn (*d*^10^) tetrapyrroles to avoid complications associated with axial ligation and the presence of π to *d* charge transfer and *d-d* excited states^89,90^ in open *d*-shell species. Apart from **P**, **Pc**, and **SubPc,** we also included other systems, presenting only one (or two, in the case of contracted systems) of the above-mentioned modifications with respect to **P**: porphyrazine (**Pz**), tetrabenzoporphyrin (**TBP**), Zn-subporphyrin (**ZnSubP**), subporphyrin (**SubP**), subporphyrazine (**SubPz**), and tribenzosubporphyrin (**TBSubP**). The study focuses on the changes (*i*)–(*iii*), mentioned above. However, for **SubP**, we also tested the role of the central coordination (*iv*), by considering the **ZnSubP** system, to assess whether the central element affects the aromaticity and whether the comparison between tri- and tetra-pyrrole/isoindoles is consistent. To determine structural changes in the physical properties, we compared the computational and experimental UV–vis spectra and analyzed the , H–L gap, and aromaticity of the different molecules.

### UV–Vis Absorption Spectra, H–L Gaps, and 

A detailed assignment of the Q and B bands has been done through TDDFT and UV–vis spectroscopy. Table 1 presents the vertical absorption energies, oscillator strengths, and TDDFT roots associated with Q and Soret (B) bands for the eight (sub)porphyrinoids under study. For the sake of simplicity, in the ensuing discussion, we will refer to the set of **P**, **Pz**, **TBP**, and **Pc** as phthalocyanines and **ZnSubP**, **SubP**, **SubPz**, **TBSubP**, and **SubPc** as subphthalocyanines. Computational absorption maxima (λ_max_, in nm) are slightly underestimated compared to those of the experimental counterpart. However, they follow the same trend, presenting excellent linear correlations with *R*^2^ = 0.98 and 0.94 for the Q and B bands, respectively (see Figure S3). While CAM-B3LYP may not reproduce the absolute experimental excitation energies to the highest accuracy, it exhibits consistency in predicting qualitative band shifts. Indeed, the relative band shifts, defined as Δλ_max_ = λ_max,X_ – λ_max,P_ (where X represents any system but **P**), show a minimal discrepancy with the experimental values, with differences not exceeding 0.2 eV (26.6 nm). For this reason, further discussions will focus on CAM-B3LYP values.

**Table 1:** Comparison of Computational and Experimental Vertical Absorption Spectra for **P**, **Pz**, **TBP**, **Pc**, **SubP**, **SubPz**, **TBSubP**, and SubPc^a^

	Q-band				Soret (B) band			
	state	λ_max_	*f*	λ_max_ exp.	state	λ_max_	*f*	λ_max_ exp.
**P**	S_1_, S_2_	520.3	0.010	565^b^	S_3_, S_4_	354.1	1.370	398^b^
**Pz**	S_1_, S_2_	537.9	0.316	596^c^	S_6_, S_7_	323.2	0.226	343^c^
**TBP**	S_1_, S_2_	582.9	0.304	623^b^	S_3_, S_4_	368.1	1.603	422^b^
**Pc**	S_1_, S_2_	636.3	0.675	671^c^	S_12_, S_13_	297.0	1.139	348^c^
**SubP**	S_1_, S_2_	403.9	0.025	454^d^	S_3_, S_4_	304.2	0.855	341^d^
**SubPz**	S_1_, S_2_	425.7	0.221	497^c^	S_9_, S_10_	262.7	0.458	290^c^
**TBSubP**	S_1_, S_2_	461.0	0.308	514^e^	S_3_, S_4_	312.4	1.055	355^e^
**SubPc**	S_1_, S_2_	503.3	0.475	565^c^	S_10_, S_11_	262.9	0.866	305^c^

a TDDFT Q and Soret (B) bands, computed absorption maxima (λ_max_ in nm), computed oscillator strengths (*f*), and experimental λ_max_. Computational results were obtained considering the same solvent as that used in experiments.

b In ethanol from ref (129).

c These results were generated by our own experimental setup in THF. In the case of **SubPz**, the spectrum corresponds to the β-substituted **SubPz** (see Figure S2a).

d In dichloromethane (DCM) from ref (95).

e In dichloromethane (DCM) from ref (128).

The Q and B band shifts of the substituted subphthalocyanines (**SubPz**, **TBSubP**, and **SubPc)** with respect to **SubP** exhibit the same behavior as the shifts observed in their phthalocyanine counterparts (**Pz**, **TBP**, and **Pc**) when compared to those of **P**. In fact, the magnitude of these band shifts is comparable between the analogs of both groups, indicating a consistent pattern in the response to substitution. The sole exception to this rule is **TBP,** where the B-band is slightly red-shifted owing to the destabilizing influence of the fused benzene in the a_1u_ (H) orbital, coupled with the absence of stabilization in the a_2u_ (H – 1) orbital due to the presence of CH–*meso* groups. The latter similarities between phthalocyanines and subphthalocyanines suggest the modifications in *meso* and β positions have a similar effect regardless of the molecule’s planarity and number of pyrrole or isoindole units. In general, the reduction in the number of pyrrole or isoindole units increases the band gap, as expected from the decrease of the π-conjugated units (the same happens with linear paraphenylenes, for instance).^91^

The optical spectra of **P** and **SubP** are primarily influenced by frontier orbitals. In these molecules, the Q-band is associated with the a_1u_ → e_g_ (in **P**) and a_2_ → e (in **SubP**) transitions. For the B-band, a_2u_ → e_g_ (**P**) and a_1_ → e (**SubP**) transitions play a major role. In general, an increase in  and  (or  and  in C_3*v*_ systems) leads to higher absorption energies in the Q and B bands, respectively. This relationship between the frontier orbitals and the absorption spectra aligns with the Gouterman model and studies by Belosludov,^43^ Martynov and Mack,^92,93^ and Nemykin.^94^ There is a particularly good correlation between the Q-band and  (), as displayed in Figure 3. It is worth noting that for **SubP**, the energy gap  of 5.7 eV at the CAM-B3LYP/cc-pVTZ level should be compared with a value of 3.64 eV obtained using B3LYP/6-311G(d).^95^ Despite the correlation between the B-band and  (), data points tend to cluster based on substitution at the *meso* positions and number of pyrrole and isoindole units, leading to a nonuniform distribution along the regression line. In the case of systems with CH–*meso*, the B and Q bands have contributions from only H – 1 (a_2u_), H (a_1u_), and L (e_g_). A similar pattern is observed for the Q-band in the N–*meso* systems. Yet the B-band in these systems not only has a significant contribution from a_2u_ to e_g_ but also incorporates transitions from b_2u_ to e_g_, see Tables S9, S11, S14, and S16. Thus, in these cases, the Gouterman model is insufficient to explain the nature of the B-band. Given the involvement of additional transitions in the B-band, we expanded the Gouterman four-orbital model to consider other orbitals involved in the transitions and their influence on the excitation energy (Section S2.3). The resulting pondered Δε shows a more uniform distribution of the data, leaving **Pz** as the only outlier (the correlation coefficient *R*^2^ increases from 0.84 to 0.98 upon removing **Pz**; see Figure S4). In **Pz**, the *E*_u_ states associated with the B_1_ and B_2_ bands show a significant contribution (the weight is 0.49; see Table S9 and Figure S5) from the b_2u_–e_g_ transition, in contrast to all other systems where the contributions come from the a_1u_ and a_2u_ to e_g_ transitions.

**Figure 2:**
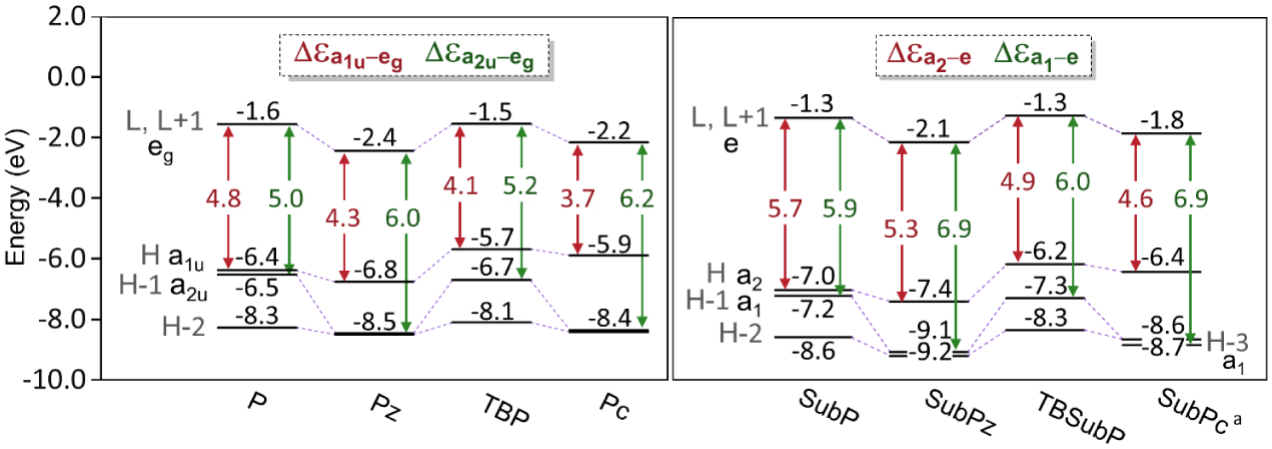
Energy of the frontier orbitals (in eV), and  and  (or  and ) (in eV) at the CAM-B3LYP/cc-pVTZ level of theory for phthalocyanines (left) and subphthalocyanines (right). In the case of **SubPc**, the orbital with a_1_ symmetry is H – 3 instead of H – 1. Further details are given in Tables S17–S19.

In **P** (**SubP**), the a_1u_ and a_2u_ (a_2_ and a_1_) orbitals correspond to H and H – 1, respectively. The latter orbitals are nearly degenerate (energy difference below 0.2 eV; see Figure 2), contributing to both the Q and B bands. Upon addition of the *benzo* substituents at the β-positions, the energy difference between a_1u_ and a_2u_ (a_2_ and a_1_) orbitals increases to approximately 1 eV, thus eliminating near degeneracy. The difference is further increased upon inclusion of the N–*meso* substituent, leading to a near degeneracy of H – 1 and H – 2 orbitals. The H–L gaps decrease with the inclusion of the *benzo* moieties, primarily due to the destabilization of the H upon addition of the substituents, which give antibonding character to this orbital around the β-position. Systems that also have N–*meso* (**Pc** and **SubPc**) exhibit further reduction of the H–L gap due to the stabilization of the LUMO orbital. This results in **Pc** having the smallest H–L gap among all the systems.

Given the similarity between the orbital distribution of S_1_ and T_1_ states, in the following, we analyze whether the Gouterman model, which is only useful to predict the Q-band, can also be used to anticipate the behavior of T_1_ and its influence in the . To this end, we have computed the triplet vertical excited states at the TDDFT level of theory (see Table S21 and Figures S6 and S7a for a comparison with TDA, Supporting Information). In all cases, the first two roots correspond to two degenerate triplet states (T_1_) with a predominant H – 1, H → L (a_2u_, a_1u_ to e_g_) transitions and energies 1.04–1.55 eV below the singlet excited states associated with the Q-band. In the case of **P** and **SubP**, two additional degenerate triplets (T_2_) exist, 0.23 and 0.44 eV beneath the first singlet excited state, respectively. For the remaining systems, T_2_ is above S_1_; however, there is an inverse relationship between the energies of T_1_ and T_2_ within each family, phthalocyanines and subphthalocyanines (when T_1_ increases, T_2_ decreases, and the other way around; see Figure S7c). T_1_ presents a positive correlation (see Figure S7b,d) with both the energy of S_1_ and  (or ). Thus, the observations made earlier in the paper regarding the role of frontier orbitals on the Q-band can be qualitatively extended to the T_1_ state.

Our analysis reveals that the evaluation of  and  do not comprehensively describe the absorption spectra, in particular the B-band for N–*meso* systems where the Gouterman model falls short. An extended Gouterman model provides a rationale for the trends observed in the B-band but lacks the simplicity of the original model. To provide a more chemically intuitive explanation, we resort to the study of the aromaticity of these compounds.

### Aromaticity of Phthalocyanines and Subphthalocyanines

The aromatic stabilization energy (ASE) is known to diminish with an increase in the size of [n]annulenes, a trend that is accompanied by a marked decrease in electron conjugation. Notably, the ASE value for [18]annulene is as low as 2.6 kcal/mol,^64^ which stands in stark contrast to that of benzene, approximately 30 kcal/mol,^96^ depending on the homodesmotic reaction considered. The local aromaticity of the pyrrole rings is important to explain the overall ASE in porphyrinoids.^97^ In our case, systems containing *benzo* rings have a multicenter index (MCI) value close to 0.050, not far from the value obtained for benzene at the same level of theory (MCI = 0.071), while all the five-membered rings display MCI values about half the values of pyrrole or lower (*benzo*-substituted compounds) (see Table S32). Nevertheless, the global aromatic character of the molecule is influenced by the conjugated pathways along the whole molecule, which pass through these five- and six-membered rings. In simple neutral porphyrinoid systems, the aromatic character expected from straightforward π-electron counting rules is observed, whereas more intricate systems call for a more profound analysis. Hence, in this study, we opt for various electronic and magnetic aromaticity measures.^51,71^

Among the few aromaticity studies of (sub)phthalocyanines, the use of magnetic criteria, especially NICS, is prevalent.^98^ However, the utilization of global NICS measures for systems featuring fused rings has, until now, limited the ability to explore conjugated pathways individually. Additionally, previous studies have not delved into the effect of molecular substitutions. In this work, we embark on a comprehensive, two-pronged exploration of aromaticity. This entails an examination of intrinsic electronic aromaticity measures such as AV1245 (and AV_min_)^82^ and electron density of delocalized bonds (EDDB),^99,100^ complemented by a comprehensive analysis of response aromaticity through the investigation of ring currents.

**Figure 3:**
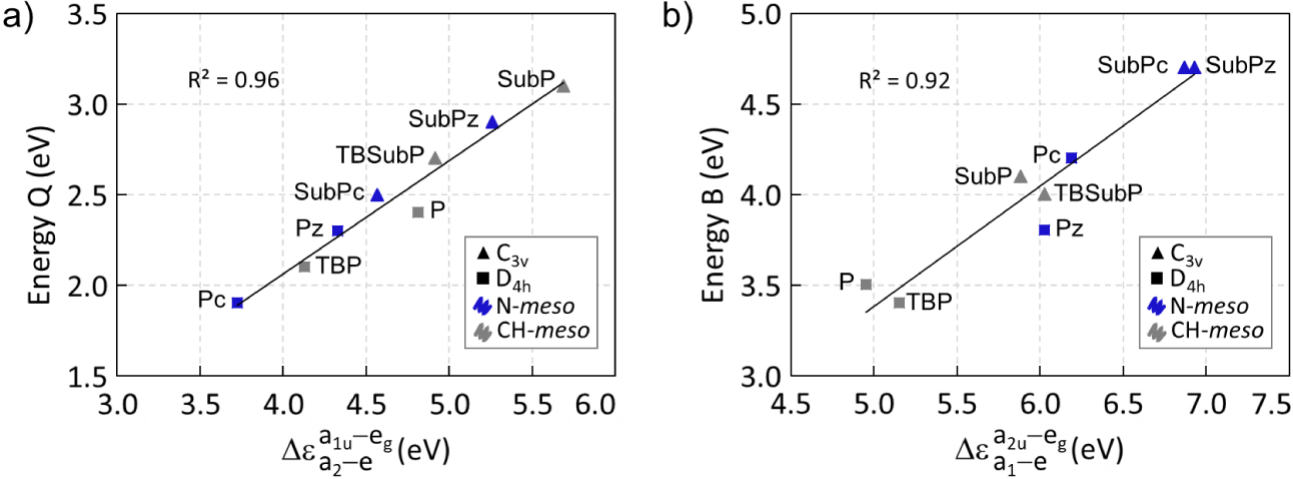
Relationship between (a) Q-band energy and  () and (b) B-band energy and  ().

From the ring current perspective, all systems display diatropic currents with global current strengths exceeding 20 nA·T^–1^, a value larger than the 12 nA·T^–1^ found in benzene (see Figure S10). Hence, all investigated systems display conjugated pathways and can be considered magnetically global aromatic (Figure 3).

**Figure 4:**
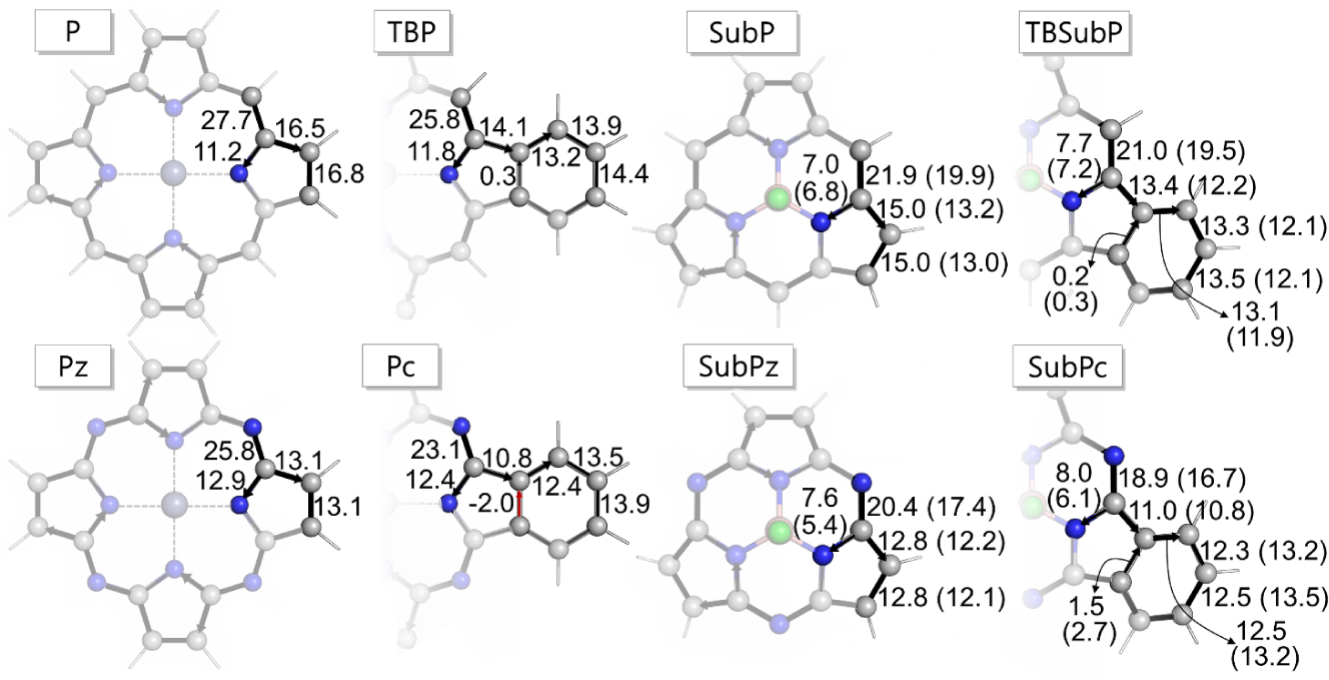
Net current strengths (in nA·T^–1^) passing through selected bonds in the S_0_ state. In the case of subphthalocyanines, the values within parentheses represent the calculated current strengths when an external magnetic field is oriented perpendicular to the plane defined by the pyrrole or isoindole ring (refer to Section S4.1 for details).

Although the current density maps show a global diatropic circulation, indicative of aromaticity, the global current is evenly split between the *outer* (o), *inner* (i), and *benzo* (b) pathways (see Scheme 2), as evidenced by examining the current strength in Figure 4. For instance, consider the case of **Pc**, where the total current of 23.1 nA·T^–1^ is broken down into two components: a 12.4 nA·T^–1^ current passing through the iii(i) pathway and a 10.8 nA·T^–1^ current passing through the ooo(o) pathway. This pattern is akin to what has been observed in free-base **Pc** and **TBP**. Both N–*meso* and *benzo* substitutions play a role in enhancing the intensity of the inner current, with the N–*meso* substitution exerting a particularly significant influence. This becomes apparent when examining the current strengths across different systems, such as the transition from **P** to **Pz**, where a clear outer pathway dominance over the inner one shifts to a similar preference upon N–*meso* substitution. Similarly, in the transition from **Pz** to **Pc**, the preference from the outer to inner pathway is entirely reversed upon *benzo* substitution. Similar trends are observed in the case of subphthalocyanines. However, due to the nonplanar nature of these molecules, defining an external magnetic field perpendicular to the system is not straightforward, and the results should be considered semiquantitative.

In the following, we examine electronic aromaticity indices, specifically AV1245 (and AV_min_), and EDDB_P_ (and limit of EDDB_P_), which provide information about the electron delocalization in the conjugated pathways of the molecule in the absence of an external perturbation.^47,48,51,63,68,71−73,101^ Unlike the magnetic indices, the nonplanarity of subphthalocyanines does not represent a challenge for electronic indices, which can also be decomposed into contributions from individual groups or fragments. AV1245 is calculated as the average of multiple four-center MCI computed at positions 1, 2, 4, and 5 for each five-atom fragment along the conjugated pathway. In contrast, AV_min_ represents the smallest absolute value among these 4-center MCI values. The EDDB method involves the decomposition of the electron density into three components: electron density localized on the atoms (EDLA), electron density localized on the bonds (EDLB), and delocalized density, referred to as the electron density of delocalized bonds (EDDB). The latter quantity, when measured within a closed circuit, serves as an indicator of aromaticity. In our study, we focus on the EDDB_P_(r) function and electron populations (referred to as EDDB_P_), which specifically consider adjacent chemical bonds along the selected pathway. Additionally, similar to the AV_min_ index, we examine the limit of EDDB_P_ (limit_EDDB_), which corresponds to the atom in the pathway with the smallest delocalized electron population. For all these indices, large values indicate aromaticity, while small values indicate nonaromaticity or antiaromaticity. AV1245 and EDDB_P_ consider the *average* delocalization along the pathways and are expected to reflect features connected with the conjugated nature of these molecules, whereas AV_min_ and lim_EDDB_ indicate the *least* delocalized fragment/atom in the pathway, and this limiting value has been successfully connected to the aromaticity of the pathway in porphyrinoids.^51,71,72^ In practice, both values contribute to the overall assessment of the conjugated pathways. Finally, in the case of **P**, we have studied the effect of a coordinated solvent molecule to Zn(II) in the aromaticity of the macrocycle. Results show only minor changes in electronic indices (Figure S21 and Table S35).

**Figure 5:**
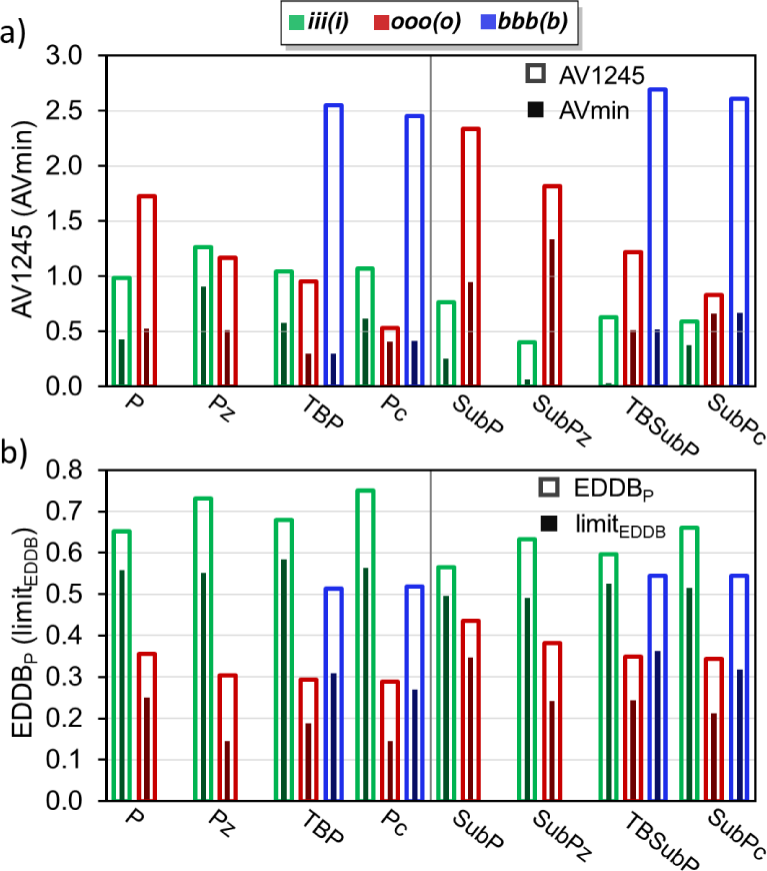
Aromaticity values of iii(i), ooo(o), and bbb(b) circuits in each system according to (a) AV1245 and (b) EDDB_P_ (normalized according to the number of atoms in the circuit) aromaticity measures. The darker filled bars represent the (a) AV_min_ and (b) limit of EDDB_P_.

The total number of nonequivalent conjugated pathways depends on the symmetry of the molecules. We identify 4 in **SubP** and **SubPz**, 6 in **P** and **Pz**, 10 in **TBSubP** and **SubPc**, and 21 in **TBP** and **Pc**, for which we have listed all electronic aromaticity indices in Tables S23–S31. However, in practice, the most important contributions are given by three conjugated pathways: *inner* iii(i), *outer* ooo(o), or *benzo* bbb (b) pathways, the results of which are summarized in Figure 5. AV1245 and AV_min_ show significantly reduced values for the most conjugated pathways in the molecules depicted in Scheme 1. Specifically, the AV1245 values are found to be below 3.0, and AV_min_ values are below 1.5 for all of the pathways. The latter figures are markedly lower than the values of 10.50 for both indices in benzene; however, they are in line with the values reported for other porphyrinoid systems.^51,71^ AV1245 and AV_min_ values do not agree on which pathway is the most conjugated, indicating that minimal and average multicenter delocalizations differ significantly. This difference is particularly evident for *benzo*-substituted molecules. As discussed in previous publications,^83,102^ AV_min_ is the index that better reflects aromatic character, whereas AV1245 provides an average delocalization value that can obscure weakly conjugated fragments.

According to EDDB_P_ and limit_EDDB_, iii(i) is always the most aromatic pathway with values lower than 0.75 and 0.59 electrons, respectively. In comparison, benzene demonstrates values of 0.92 electrons for both EDDB_P_ and limit_EDDB_. Hence, there is a qualitative consensus regarding the most conjugated pathway in phthalocyanines, with AV_min_, EDDB_P_, and limit_EDDB_ consistently identifying the iii(i) pathway as the most conjugated. The sole exception is observed in **Pz**, where AV_min_ does not show a distinct preference between the ooo(o) and iii(i) pathways. Conversely, AV_min_ identifies the ooo(o) and bbb(b) as the most aromatic pathways in subphthalocyanines, whereas limit_EDDB_ always identifies the iiii(i) pathway as the most aromatic. Nevertheless, in instances where the aromaticity of the ooo(o) and iii(i) pathways is ranked separately for each molecule using AV1245 and EDDB_P_, both indices consistently produce the same order, from the most aromatic to the least aromatic. The only deviation occurs in the ranking of the iii(i) circuit in subphthalocyanines, which is also observed with the electronic-based FLU and geometric-based HOMA indices (Tables S23–S31). In the case of the ooo(o) pathway, there is even a good linear correlation between AV1245 and EDDB_P_ (see Figure S20). All in all, the most important difference between the electronic indices and magnetic ring currents is the magnitude of the aromaticity. According to the ring current strengths, all compounds are highly aromatic, while electronic indices indicate lower aromaticity.

**Figure 6:**
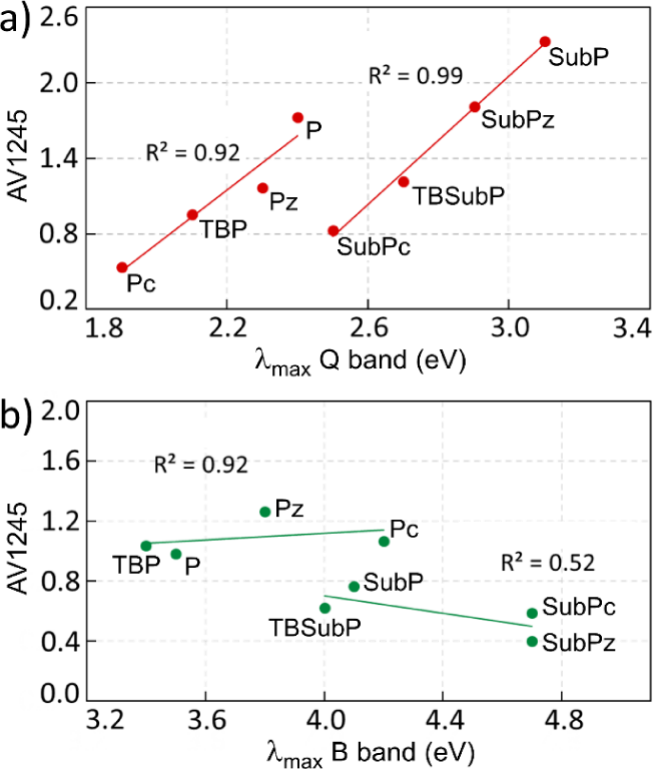
Relationship between (a) Q-band energy and AV1245 of the ooo(o) pathway and (b) B-band energy and AV1245 of the iii(i) pathway.

The aromaticity of each pathway reflects the trends we observed in the UV–vis absorption energies (Figure 6). Both AV1245 and EDDB_P_ show that the aromaticity of the ooo(o) circuit decreases along the **P**–**Pz**–**TBP**–**Pc** and **SubP**–**SubPz**–**TBSubP**–**SubPc** series, as occurred for the energy of the Q-band and . All aromaticity indices uniformly recognize the ooo(o) circuit in phthalocyanines as being less aromatic than the corresponding ooo(o) pathway in their contracted analogs, namely subphthalocyanines. This observation aligns with the higher excitation energies observed in the Q-band of these compounds.

AV_min_ and limit_EDDB_, while they might reflect the limiting conjugated part of the pathway, do not show any evident connection with the Q and B bands. Instead, they can be used to identify the least conjugated fragment in the molecule and be instrumental in modifying the pathway’s aromaticity and, given their connection to the average counterparts, in the case of the ooo(o) pathway, tuning the Q-band. In Figures S16–S19, we split the information on AV1245 into five-atom fragments, in which we can easily recognize the fragment(s) giving rise to AV_min_. Interestingly, the values of the fragments in the ooo(o) pathways follow the same distribution for a given phthalocyanine and its analog subphthalocyanine. The least (or second least) delocalized fragment of the ooo(o) pathways always corresponds to the fragment centered in the *meso*-position. This implies that *meso*-substitution influences the Q bands across all systems studied. To achieve a blue shift of the Q bands, a *meso*-substitution is necessary, but it must be distinct from the N–*meso* substitution. Indeed, the transition from **P** to **Pz** or **Pc** (N–*meso* substitution) results in a reduced AV1245 value, which in turn shifts the Q-band to the red. The addition of a *benzo*-group affects similarly, reducing the delocalization of other five-atom fragments and red-shifting the Q-band. The interplay of these effects results in a 3-fold reduction in delocalization for certain fragments in phthalocyanines and a 5-fold reduction in subphthalocyanines. This significant decrease in delocalization effectively eliminates any conjugated fragments within the ooo(o) pathway, consequently leading to a red shift in the Q bands. While the aforementioned analysis provides a reasonable understanding of the Q-band shifts, the correlation between the B-band and AV1245 values is less clear (see Figure 6b).

## Conclusions

This study examines substituted phthalocyanines (**P**, **TBP**, **Pc**, and **Pz**) and their contracted analogs, subphthalocyanines (**SubP**, **TBSubP**, **SubPc**, and **SubPz**), which are characterized by nonplanar, bowl-shaped geometries. The methodology employed in this study, which includes CAM-B3LYP/cc-pVTZ calculations, is validated through the comparison of UV–vis computational and experimental studies. In addition, we rely on the Gouterman four-orbital model for porphyrinoids.

Our analysis reveals that evaluation of the four orbital energies is insufficient to describe the absorption spectra, particularly the B-band for N–*meso* systems, where deviations from the Gouterman model are apparent. An extension of the Gouterman model, including more orbitals, provides a rationale for the trends observed in the B-band, but it sacrifices the simplicity of the original model. Consequently, we turn to the examination of the aromaticity of these compounds, providing a more chemically intuitive explanation of their spectral features.

Magnetic response indices characterize all the molecules studied as aromatic, exhibiting important ring current strengths. Conversely, an analysis of electron delocalization and π-conjugation through AV1245, AV_min_, and EDDB indices reveals that—despite the important response upon the application of an external magnetic field—the conjugated circuits are much less aromatic than those found in classical organic molecules like benzene, being closer to those already reported in other porphyrinoid systems.^47,71^ This evidence adds to the results already reported in the literature, where intrinsic (electronic) and response (magnetic) measures of aromaticity do not align.^72,73,101,103−109^ This divergence between intrinsic electronic and magnetic aromaticity measures adds a critical dimension to our understanding of these complexes, offering insights relevant to inorganic chemists focused on the interplay between electronic structure and reactivity in coordination compounds. In general, subphthalocyanines can be considered slightly more aromatic than phthalocyanines, according to the least delocalized fragment of the external-most circuit of these systems, and in agreement with the larger HOMO–LUMO gap observed in subphthalocyanines.

Interestingly, the electronic aromaticity indices help explain part of the UV–vis spectrum of (sub)phthalocyanines, giving a direct connection between the aromaticity of the external-most conjugated pathways and the Q bands. Particularly, the substitution at the *meso* position seems to have a large effect on the aromaticity and the position of the Q and B bands. This insight is pivotal for pinpointing modifications in porphyrinoid structures that lead to marked shifts in the UV–vis bands. Our findings offer a strategic framework for designing novel phthalocyanine derivatives, where the fine-tuning of electronic properties through structural modifications can lead to the development of advanced materials and catalysts.

## Computational Details

The systems presented in Scheme 1 have been fully optimized and characterized as energy minima in the ground state using harmonic vibrational frequency calculations at the CAM-B3LYP/cc-pVTZ level of theory.^110,111^ The choice of the functional was based on the comparison between optimized **Pc** and **SubPc** using B3LYP, ωB97X, M062X, TPSSH, and LC-ωHPBE together with cc-pVTZ basis set and the X-ray structure (Tables S1–S4). Additionally, we examined the UV–vis spectra using different functionals: B3LYP, CAM-B3LYP, ωB97xD, M062X, and LC-BLYP and optimally tuned LC-BLYP functionals (Tables S5–S7) to assess the performance of each method. We computed in-solution optical spectra of all systems using CAM-B3LYP functional and cc-pVTZ basis set by means of TDDFT considering the first 20 singlet states solvated in THF, DCM, or ethanol, according to the experimental data available. The effect of the implicit solvent has been accounted with the polarizable continuum model (PCM)^112^ approach. For the calculation of the vertical triplet state, our choice was to employ time dependent and Tamm–Dancoff approximation (TD and TDA)-DFT (CAM-B3LYP/cc-pVTZ) to calculate the two degenerate triplets resulting from having degenerate L/L + 1 orbitals. All calculations have been done with the Gaussian 09 and 16 software packages.^114^ For the characterization of the aromaticity, we used a variety of measures, including geometrical, electronic, and magnetic indices, to determine local and global aromaticity. The 2- (delocalization indices (DIs)), 3-, and 4-center indices for each set of atoms in the system and the fluctuation index (FLU),^113,115^ bond order alternation (BOA),^63^ I_ring_,^116^ multicenter index (MCI),^117^ AV1245,^82^ and AV_min_^102^ electronic indices were computed using AIMAll^118^ and ESI-3D^113,119,120^ (available upon request: ematito@gmail.com) programs. The harmonic oscillator measure of aromaticity (HOMA)^121^ and the bond length alternation (BLA) were calculated with ESI-3D using molecular geometries as input.^64^ Electron density of delocalized bond (EDDB)^99,100^ results were computed using NBO 6.0^122^ software to first obtain the natural atomic orbitals (NAO) and the 1-electron density matrix used as input for the RunEDDB (v20200925) program (available on www.aromaticity.eu). Finally, the magnetic current density and the current strengths were obtained using Gaussian 09 together with the GIMIC program.^123,124^ Further explanation regarding the calculation of aromaticity indices can be found, Sections S1 and S4.

## Experimental Data

**SubPc**,^125^**Pz**,^126^ and **SubPz**(127) were synthesized following reported procedures. **Pc** was purchased from Aldrich and used without further purification. The UV–vis spectra of these compounds were recorded in THF (concentration = 2 × 10^–5^ M) employing a JASCO-V660 spectrophotometer. **SubPz** has to be prepared as β*-*substituted-**SubPz** with propyl groups for synthetic reasons.^11^ However, since there is no conjugation of the ethyl groups with the pyrroles, their influence on the absorption spectrum is expected to be minor (Q-band can be displaced 5–10 nm at most).^11^ The data for the remaining molecules have been sourced from the literature. The measured absorption spectra of **SubP** and **TBSubP** were obtained in DCM using derivatives bearing OMe and OH as axial ligands, respectively, in order to avoid hydrolysis.^95,128^ It is well-known that neither the shape nor displacement of the spectrum is highly affected by the axial ligand. In the case of **P** and **TBP**, presenting very poor solubility, the absorption spectra were obtained in ethanol.^129^

## Data Availability

The inputs and outputs of the calculations, including Cartesian coordinates (CML), are available in ioChem-BD^130^ and can be accessed via https://doi.org/10.19061/iochem-bd-4-71.

## Supporting Information Available

Detailed computational data including performance assessments of various functionals on geometry optimization, experimental and computational UV–vis absorption spectra, and electron delocalization and aromaticity analyses (PDF)
