# Supplementary material for: From (Sub)Porphyrins to (Sub)Phthalocyanines: Aromaticity Signatures in the UV–Vis Absorption Spectra
Source: Inorg Chem. 2024 Sep 19;63(39):18251–62. doi: 10.1021/acs.inorgchem.4c03139 (PMC11465665; doi:10.1021/acs.inorgchem.4c03139)
Supplement: Supplementary file 1 — ic4c03139_si_001.pdf [file ic4c03139_si_001.pdf]

Supporting Information: From (Sub)Porphyrins to (Sub)Phthalocyanines: Aromaticity Signatures in the UV-Vis Absorption Spectra

Sílvia Escayola,[a,b] Jorge Labella,[c] Dariusz W. Szczepanik,[d] Albert Poater,[a] Tomas Torres,[c,e,f] Miquel Solà,*[a] Eduard Matito*[b,g]

[a]*Institut de Química Computacional i Catàlisi and Departament de Química, Universitat de Girona, C/ Maria Aurèlia Capmany, 69, 17003 Girona, Catalonia, Spain.*

[b]*Donostia International Physics Center (DIPC), Donostia, Euskadi, Spain.*

[c]*Departamento de Química Orgánica, Universidad Autónoma de Madrid, Madrid 28049, Spain.*

[d]*Department of Theoretical Chemistry, Faculty of Chemistry, Jagiellonian University, 30-387 Kraków, Poland.*

[e]*Institute for Advanced Research in Chemical Sciences (IAdChem), Universidad Autónoma de Madrid, Madrid, 28049, Spain.*

[f]*IMDEA-Nanociencia, Campus de Cantoblanco, Madrid, 28049, Spain.*

[g]*Ikerbasque Foundation for Science, 48011 Bilbao, Euskadi, Spain.*

emails: miquel.sola@udg.edu; ematito@gmail.com

Contents

S1. Performance of Different Functionals 2

S2. UV-Vis Spectra and Frontier Molecular Orbitals 12

S2.1 Experimental UV-Vis absorption spectra 12

S2.2 Computational UV-Vis absorption spectra and molecular orbitals 13

S2.3 Expanded Gouterman model to interpret B band 25

S3. Singlet-Triplet Energy Gap 27

S4. Electron Delocalization and Aromaticity Measures 29

S4.1. GIMIC 29

S4.2. HOMA, FLU, AV1245, AV_{min}, and EDDB 33

S4.3. N-Center Delocalization Indices (δ_{2c} and δ_{4c}) 40

S4.4. Solvent effects 44

S5. References 45

S1. Computational details

S1.1. Performance of Different Functionals

In order to assess the density functional approximations (DFA) we are employing to obtain optimized geometries, ultraviolet-visible (UV-Vis) absorption spectra, and aromaticity studies, we evaluated the performance of several DFAs and compared them against the experimental data available. First, we compared X-Ray crystal structures of Phthalocyanine (**Pc**)[1] and subphthalocyanine (**SubPc**)[2] [CCDC reference: PHTLBC][3] with the values obtained using B3LYP[4-6], CAM-B3LYP[7], w-B97X[8], M06-2X[9], TPSSh[10], and LC-ωhPBE[11,12] functionals together with the cc-pVTZ[13] basis set.

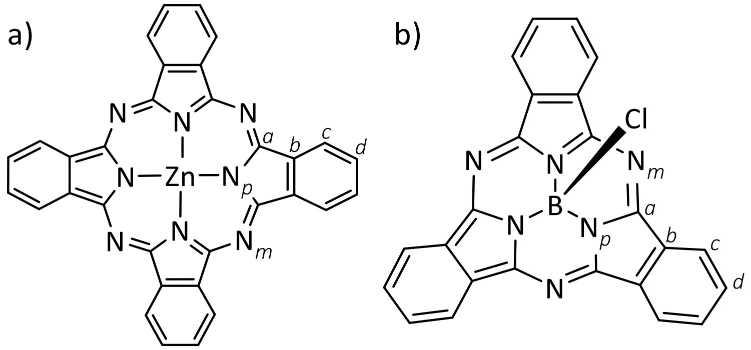

Figure S1. Atom labels in a) **Pc** and b) **SubPc** systems.

The DFAs that better reproduce the X-Ray structure are B3LYP followed by CAM-B3LYP and TPSSh, with pretty similar mean absolute errors (MAE) in the various parameters (bond lengths, angles and P1 and P2 distances), see Tables S1-4.

Table S1. Experimental vs. computed geometries of **Pc** using different functionals. Distances in Å and angles in degrees.

	Zn−N$_p$	Zn−N$_p$−C$_a$	N$_p$−Zn−N$_p$	N$_p$−C$_a$−N$_m$	C$_a$−N$_p$−C$_a$	C$_a$−N$_m$−C$_a$	C$_a$−N$_p$	C$_a$−N$_m$	C$_a$−C$_b$	C$_b$−C$_b$	C$_b$−C$_c$	C$_c$−C$_d$	C$_d$−C$_d$
X-Ray	1.980	125.4	90.0	127.8	109.1	123.5	1.369	1.331	1.455	1.400	1.393	1.391	1.396
B3LYP	2.000	125.0	90.0	127.4	110.0	125.1	1.367	1.326	1.459	1.406	1.390	1.388	1.402
CAM-B3LYP	1.988	125.0	90.0	127.4	109.9	125.1	1.360	1.319	1.456	1.396	1.385	1.381	1.398
w-B97X	1.989	125.0	90.0	127.6	110.0	124.8	1.360	1.322	1.459	1.395	1.388	1.383	1.401
M06-2X	1.997	124.9	90.0	127.6	110.2	124.9	1.361	1.324	1.460	1.399	1.387	1.385	1.401
TPSSh	1.993	125.2	90.0	127.5	109.6	124.6	1.370	1.328	1.457	1.407	1.392	1.390	1.403
LC-ωhPBE	1.980	125.1	90.0	127.5	109.8	124.7	1.355	1.317	1.456	1.389	1.384	1.378	1.397

Table S2. Experimental vs. computed geometries of **SubPc** using different functionals. Distances in Å and angles in degrees.

	B−X	B−N$_p$	N$_p$−B−N$_p$	N$_p$−B−X	C$_a$−N$_p$	C$_a$−N$_m$	C$_a$−C$_b$	C$_b$−C$_b$	C$_b$−C$_c$	C$_c$−C$_d$	C$_d$−C$_d$	P1[a]	P2[b]
X-Ray	1.863	1.467	105.2	113.3	1.369	1.344	1.460	1.427	1.391	1.370	1.394	0.58	2.47
B3LYP	1.879	1.486	105.1	113.6	1.367	1.336	1.453	1.427	1.391	1.386	1.403	0.59	2.57
CAM-B3LYP	1.863	1.482	104.8	113.8	1.357	1.330	1.451	1.416	1.387	1.379	1.398	0.60	2.57
w-B97X	1.853	1.487	104.5	114.1	1.357	1.332	1.454	1.414	1.391	1.380	1.402	0.61	2.60
M06-2X	1.854	1.486	104.6	114.0	1.359	1.333	1.454	1.418	1.390	1.382	1.402	0.60	2.60
TPSSh	1.868	1.488	104.8	113.8	1.366	1.339	1.452	1.428	1.393	1.388	1.404	0.60	2.57
LC-ωhPBE	1.841	1.483	104.3	114.3	1.352	1.327	1.452	1.408	1.387	1.375	1.398	0.61	2.58

[a]Distance between the B atom and the plane defined by the 3 N$_p$ atoms. [b]Bowl-depth: distance between the B atom and the mean plane defined by the 6 C$_d$ atoms.

Table S3. Absolute error in absolute values with respect the experimental values in **Pc** and **SubPc**. Distances in Å and angles in degrees.

	Phthalocyanine (**Pc**)												
	Zn−N$_p$	Zn−N$_p$−C$_a$	N$_p$−Zn−N$_p$	N$_p$−C$_a$−N$_m$	C$_a$−N$_p$−C$_a$	C$_a$−N$_m$−C$_a$	C$_a$−N$_p$	C$_a$−N$_m$	C$_a$−C$_b$	C$_b$−C$_b$	C$_b$−C$_c$	C$_c$−C$_d$	C$_d$−C$_d$
X-Ray	0.000	0.000	0.0	0.0	0.0	0.0	0.000	0.000	0.000	0.000	0.000	0.000	0.000
B3LYP	0.020	0.408	0.0	0.4	0.9	1.6	0.002	0.005	0.004	0.006	0.003	0.003	0.006
CAM-B3LYP	0.008	0.374	0.0	0.4	0.8	1.6	0.009	0.012	0.001	0.004	0.008	0.010	0.002
w-B97X	0.009	0.375	0.0	0.2	0.9	1.3	0.009	0.009	0.004	0.005	0.005	0.008	0.005
M06-2X	0.017	0.495	0.0	0.2	1.1	1.4	0.008	0.007	0.005	0.001	0.006	0.006	0.005
TPSSh	0.013	0.208	0.0	0.3	0.5	1.1	0.001	0.003	0.002	0.007	0.001	0.001	0.007
LC-ωhPBE	0.000	0.317	0.0	0.3	0.7	1.2	0.014	0.014	0.001	0.011	0.009	0.013	0.001

SubPhthalocyanine (**SubPc**)													
	B−X	B−N_p	N_p−B−N_p	N_p−B−X	C_a−C_b	C_a−N_p	C_a−N_m	C_b−C_b	C_b−C_c	C_c−C_d	C_d−C_d	P1[a]	P2[b]
X-Ray	0.000	0.000	0.0	0.0	0.000	0.000	0.000	0.000	0.000	0.000	0.000	0.00	0.00
B3LYP	0.016	0.019	0.1	0.3	0.002	0.008	0.007	0.000	0.000	0.016	0.009	0.01	0.10
CAM-B3LYP	0.000	0.015	0.4	0.5	0.012	0.014	0.009	0.011	0.004	0.009	0.004	0.02	0.10
w-B97X	0.010	0.020	0.7	0.8	0.012	0.012	0.006	0.013	0.000	0.010	0.008	0.02	0.13
M06-2X	0.009	0.019	0.6	0.7	0.010	0.011	0.006	0.009	0.001	0.012	0.008	0.02	0.13
TPSSh	0.005	0.021	0.4	0.5	0.003	0.005	0.008	0.001	0.002	0.018	0.010	0.02	0.10
LC-ωhPBE	0.022	0.016	0.9	1.0	0.017	0.017	0.008	0.019	0.004	0.005	0.004	0.03	0.11

[a]Distance between the B atom and the plane defined by the 3 N_p atoms. [b]Bowl-depth: distance between the B atom and the mean plane defined by the 6 C_d atoms.

Table S4. Mean absolute error (MAE) for the bond distances (r_{ij}), angles (α) and P1[a] and P2[b] distances with respect the experimental values in **Pc** and **SubPc**. Distances in Å and angles in degrees.

	MAE r_{ij} **Pc**	MAE α **Pc**	MAE r_{ij} **SubPc**	MAE α **SubPc**	MAE P1[a] P2[b] **SubPc**
B3LYP	0.006	0.7	0.009	0.2	0.06
CAM-B3LYP	0.007	0.7	0.009	0.5	0.06
w-B97X	0.007	0.6	0.010	0.7	0.08
M06-2X	0.007	0.7	0.009	0.7	0.08
TPSSh	0.004	0.5	0.008	0.5	0.06
LC-ωhPBE	0.008	0.6	0.012	1.0	0.07

[a]Distance between the B atom and the plane defined by the 3 N_p atoms. [b]Bowl-depth: distance between the B atom and the mean plane defined by the 6 C_d atoms.

We also checked the effect of DFAs on the absorption spectra and HOMO-LUMO gaps. For the simulation of UV-Vis spectra, we took **Pc** and **SubPc** gas phase CAM-B3LYP/cc-pVTZ optimized geometries to compute UV-Vis spectra with five different DFAs: CAM-B3LYP, wB97xD, optimally tuned (OT) long-range corrected (LC)-BLYP, B3LYP, and M062X, in solution (scrf=(pcm,solvent=THF)), in all cases we used the cc-pVTZ basis set. The optimized attenuating parameter (ω) in OT-LC-BLYP has been obtained varying ω in order to minimize J^* while satisfying Janak's theorem to the greatest extent possible.[14]

$$J^*(\omega) = \sqrt{\left|\varepsilon^{\omega}_{HOMO}(N) + IP(N)\right|^2 + \left|\varepsilon^{\omega}_{HOMO}(N+1) + EA(N)\right|^2} \qquad (1)$$

In equation 1, IP and EA are the vertical ionization potential and the electron affinity, respectively. The optimization of ω was done without taking into account the effect of the solvent.[15] We obtained optimized ω values of 0.1819 and 0.1937 a.u.$^{-1}$ for **Pc** and **SubPc**, respectively.

The results obtained with the five functionals for the Q and B bands have been compared to the experimental values (Table 1, Figure S2 and Table S5). In all cases, we found errors below 0.3 eV (74 nm) for the Q-band and 0.7 eV (53 nm) for the B-band, which is consistent with values reported in the literature for computed spectra.[16,17,34] All DFAs overestimate the experimental energies of Q- and B-bands, with the exception of the B-band of **SubPc** that it is correctly predicted by B3LYP. Unfortunately, all methods fail in the prediction of oscillator strengths being B3LYP the closer to experimental results (the magnitude of *f* match better the relative intensity of Q and B-bands).

Table S5. Computed energies (eV), wavelength (nm), with the errors compared to experimental data shown in parenthesis, and oscillator strengths (*f*) for the Q and B bands at DFA/cc-pVTZ PCM=(THF) level of theory, for a) **Pc** and b) **SubPc**.

**a) Pc**	band	roots	*f*	energy		wavelength	
CAM-B3LYP	Q	1,2	0.675	1.9	(0.1)	636.3	(-34.7)
	B	12,13	1.139	4.2	(0.6)	297.0	(-51.0)
wB97xD	Q	1,2	0.667	1.9	(0.1)	650.0	(-21.0)
	B	12,13	0.956	4.2	(0.6)	294.8	(-53.2)
OT-LC-BLYP	Q	1,2	0.607	2.0	(0.2)	630.1	(-40.9)
	B	12,13	0.964	3.9	(0.3)	316.9	(-31.1)
B3LYP	Q	1,2	0.619	2.0	(0.2)	612.8	(-58.2)
	B	16,17	0.253	3.7	(0.1)	333.5	(-14.5)
		19,20	0.531	3.8	(0.2)	327.4	(-20.6)
M06-2X	Q	1,2	0.698	2.0	(0.2)	615.3	(-55.7)
	B	12,13	1.124	4.2	(0.6)	295.2	(-52.9)

b) **SubPc**	band	roots	*f*	energy		wavelength	
CAM-B3LYP	Q	1,2	0.475	2.5	(0.3)	503.3	(-61.7)
	B	10,11	0.866	4.7	(0.6)	262.9	(-42.1)
wB97xD	Q	1,2	0.474	2.5	(0.3)	503.9	(-61.1)
	B	10,11	0.861	4.8	(0.7)	260.4	(-44.6)
OT-LC-BLYP	Q	1,2	0.434	2.5	(0.3)	502.8	(-62.2)
	B	14,15	0.557	4.5	(0.4)	277.7	(-27.3)
B3LYP	Q	1,2	0.432	2.5	(0.3)	505.0	(-60.0)
	B	10,11	0.284	4.1	(0.0)	304.8	(-0.2)
M06-2X	Q	1,2	0.494	2.5	(0.3)	490.8	(-74.2)
	B	11,12	0.506	4.7	(0.6)	261.4	(-43.6)

Table S6. Character of the excitations corresponding to the Q and B-bands, for a) **Pc** and b) **SubPc**. The a_{2u} (a_1), a_{1u} (a_2) and e_g (e) orbitals are highlighted in bold.

**a)**	CAM-B3LYP		wB97xD		OT-LC-BLYP		B3LYP		M06-2X	
	transition	weight	transition	weight	transition	weight	transition	weight	transition	weight
Q_1	**146 → 149**	0.14	**146 → 148**	0.15	**145 → 149**	-0.15	**142 → 149**	-0.13	**145 → 149**	-0.13
	**147 → 148**	0.69	**147 → 149**	0.69	**147 → 148**	0.69	**147 → 148**	0.70	**147 → 148**	0.69
Q_2	**146 → 148**	-0.14	**146 → 149**	-0.15	**145 → 148**	0.15	**142 → 148**	0.13	**145 → 148**	0.13
	**147 → 149**	0.69	**147 → 148**	0.69	**147 → 149**	0.69	**147 → 149**	0.70	**147 → 149**	0.69
B_1	138 → 149	-0.18	139 → 149	0.13	134 → 148	-0.10	139 → 149	0.48	136 → 148	-0.10
	139 → 148	-0.14	145 → 148	-0.32	137 → 149	-0.18	**142 → 149**	0.49	139 → 149	-0.19
	145 → 149	0.20	146 → 148	0.57	138 → 148	0.13	145 → 149	0.14	140 → 148	0.15
	**146 → 149**	0.60	**147 → 149**	-0.13	144 → 149	0.25	–	–	**145 → 149**	0.60
	**147 → 148**	-0.14	–	–	**145 → 149**	0.59	–	–	146 → 149	-0.19
	136 → 149	-0.10	–	–	**147 → 148**	0.14	–	–	**147 → 148**	0.13
B_2	138 → 148	-0.18	139 → 148	0.13	134 → 149	0.10	139 → 148	0.48	136 → 149	0.10
	139 → 149	-0.14	145 → 149	0.32	137 → 148	-0.18	**142 → 148**	0.49	139 → 148	-0.19
	145 → 148	-0.20	146 → 149	0.57	138 → 149	0.13	145 → 148	-0.14	140 → 149	0.15
	**146 → 148**	0.60	**147 → 148**	0.13	144 → 148	-0.25	–	–	**145 → 148**	0.60
	**147 → 149**	0.14	–	–	**145 → 148**	0.59	–	–	146 → 148	0.19
	134 → 150	-0.12	–	–	**147 → 149**	-0.14	–	–	**147 → 149**	-0.13
B	–	–	–	–	–	–	138 → 148	0.54	–	–
	–	–	–	–	–	–	139 → 149	0.20	–	–
	–	–	–	–	–	–	**142 → 149**	-0.30	–	–
	–	–	–	–	–	–	**147 → 153**	-0.25	–	–
B	–	–	–	–	–	–	138 → 149	0.54	–	–
	–	–	–	–	–	–	139 → 148	0.20	–	–
	–	–	–	–	–	–	**142 → 148**	-0.30	–	–
	–	–	–	–	–	–	**147 → 154**	-0.25	–	–

**b)**	CAM-B3LYP		wB97xD		OT-LC-BLYP		B3LYP		M06-2X	
	transition	weight	transition	weight	transition	weight	transition	weight	transition	weight
Q_1	**107 → 112**	-0.10	**107 → 112**	-0.11	**107 → 112**	-0.11	**107 → 112**	0.10	**110 → 111**	0.70
	**110 → 111**	0.69	**110 → 111**	0.69	**110 → 111**	0.69	**110 → 111**	0.70		
Q_2	**107 → 111**	0.10	**107 → 111**	0.10	**107 → 111**	0.11	**107 → 111**	-0.10	**110 → 112**	0.70
	**110 → 112**	0.69	**110 → 112**	0.69	**110 → 112**	0.69	**110 → 112**	0.70		
B_1	104 → 111	-0.15	101→ 112	0.11	101 → 111	0.11	104 → 111	-0.19	101 → 112	-0.17
	**107 → 111**	0.62	102 → 111	0.11	104 → 112	0.45	**107 → 111**	0.66	102 → 111	-0.17
	108 → 111	-0.12	104 → 111	-0.31	105 → 111	0.10	–	–	105 → 112	0.13
	109 → 112	-0.12	**107 → 111**	0.57	106 → 112	-0.10	–	–	106 → 111	0.13
	**110 → 112**	-0.12	**110 → 112**	-0.13	**107 → 112**	-0.44	–	–	**107 → 111**	0.57
	–	–	–	–	**110 → 111**	-0.12	–	–	108 → 111	-0.11
	–	–	–	–	110 → 115	0.19	–	–	109 → 112	-0.11
B_2	104 → 112	-0.15	101 → 111	0.11	101 → 111	0.11	104 → 112	-0.19	101 → 111	-0.17
	**107 → 112**	0.62	102 → 112	-0.11	104 → 112	0.45	**107 → 112**	0.66	102 → 112	0.17
	108 → 112	0.12	104 → 112	-0.31	105 → 111	0.10	–	–	105 → 111	0.13
	109 → 111	-0.12	**107 → 112**	0.57	106 → 112	-0.10	–	–	106 → 112	-0.13
	**110 → 111**	0.12	**110 → 111**	0.13	**107 → 112**	-0.44	–	–	**107 → 112**	0.57
	–	–	–	–	**110 → 111**	-0.12	–	–	108 → 112	0.11
	–	–	–	–	110 → 115	0.19	–	–	109 → 111	-0.11
B	–	–	–	–	102 → 112	-0.11	–	–	–	–
	–	–	–	–	103 → 111	0.11	–	–	–	–
	–	–	–	–	104 → 111	0.32	–	–	–	–
	–	–	–	–	**107 → 111**	0.48	–	–	–	–
	–	–	–	–	108 → 111	-0.17	–	–	–	–
	–	–	–	–	109 → 112	-0.17	–	–	–	–
	–	–	–	–	110 → 114	0.14	–	–	–	–
B	–	–	–	–	102 → 111	-0.11	–	–	–	–
	–	–	–	–	103 → 112	-0.11	–	–	–	–
	–	–	–	–	104 → 112	0.32	–	–	–	–
	–	–	–	–	**107 → 112**	0.48	–	–	–	–
	–	–	–	–	108 → 112	0.17	–	–	–	–
	–	–	–	–	109 → 111	-0.17	–	–	–	–
	–	–	–	–	110 → 115	0.14	–	–	–	–

Table S7. Order, molecular orbital (MO) number, and energy of the relevant a_{2u} (a_1), a_{1u} (a_2) and e_g (e) orbitals in a) **Pc** (b) **SubPc**) and Δε_a_{1u}-e_g and Δε_a_{2u}-e_g (or Δε_a_2-e and Δε_a_1-e) in eV obtained with the different DFAs.

**a) Pc**	symmetry	MO no.	orbital	ε	Δε_a_{1u}-e_g	Δε_a_{2u}-e_g
CAM-B3LYP	a_{2u}	146	HOMO-1	-8.4	3.7	6.2
	a_{1u}	147	HOMO	-5.9		
	e_g	148	LUMO	-2.2		
	e_g	149	LUMO+1	-2.2		
wB97xD	a_{2u}	146	HOMO-1	-8.9	4.7	7.2
	a_{1u}	147	HOMO	-6.4		
	e_g	148	LUMO	-1.7		
	e_g	149	LUMO+1	-1.7		
OT-LC-BLYP	a_{2u}	145	HOMO-2	-8.5	4.5	6.6
	a_{1u}	147	HOMO	-6.3		
	e_g	148	LUMO	-1.9		
	e_g	149	LUMO+1	-1.9		
B3LYP	a_{2u}	142	HOMO-5	-7.2	2.2	4.1
	a_{1u}	147	HOMO	-5.2		
	e_g	148	LUMO	-3.0		
	e_g	149	LUMO+1	-3.0		
M06-2X	a_{2u}	145	HOMO-2	-8.4	3.4	5.9
	a_{1u}	147	HOMO	-6.0		
	e_g	148	LUMO	-2.5		
	e_g	149	LUMO+1	-2.5		

**b) SubPc**	symmetry	MO no.	orbital	ε	Δε_a_2-e	Δε_a_1-e
CAM-B3LYP	a_1	107	HOMO-3	-8.7	4.5	6.8
	a_2	110	HOMO	-6.5		
	e	111	LUMO	-1.9		
	e	112	LUMO+1	-1.9		
wB97xD	a_1	107	HOMO-3	-9.3	5.6	7.9
	a_2	110	HOMO	-7.0		
	e	111	LUMO	-1.4		
	e	112	LUMO+1	-1.4		
OT-LC-BLYP	a_1	107	HOMO-3	-8.9	5.3	7.4
	a_2	110	HOMO	-6.8		
	e	111	LUMO	-1.5		
	e	112	LUMO+1	-1.5		
B3LYP	a_1	107	HOMO-3	-7.4	2.7	4.6
	a_2	110	HOMO	-5.6		
	e	111	LUMO	-2.8		
	e	112	LUMO+1	-2.8		
M06-2X	a_1	107	HOMO-3	-8.8	4.2	6.5
	a_2	110	HOMO	-6.5		
	e	111	LUMO	-2.2		
	e	112	LUMO+1	-2.2		

In Tables S6 and S7, we can observe that the order of the a_{2u} orbital in **Pc** varies with the DFA being the HOMO-1 (CAM-B3LYP and wB97xD), HOMO-2 (OT-LC-BLYP and M062X) or HOMO-5 (B3LYP). However, the excitations corresponding to Q and B-bands arise mainly from the transition between a_{2u} (a_1), a_{1u} (a_2), and e_g (e) orbitals independently of its order (Table S6). Among studied functionals, B3LYP is the one presenting the most distinct results with the lowest $\Delta\varepsilon\_a_{1u}$-e_g and $\Delta\varepsilon\_a_{2u}$-e_g more than 1.5 eV lower than the rest of the functionals.

Despite being B3LYP the best DFA in reproducing the excitation energies, a previous study proved the limitations of energy benchmarks. In particular, Mg-porphyrin B3LYP provided good excitation energies but gave a poor description of the wavefunction in terms of exciton size and electron-hole correlation wavefunction descriptors. On the contrary, range-separated CAM-B3LYP functional gave poorer energies and good wavefunction.[18] Moreover, we also know from previous studies[19-21] that the use of range-separated functionals like CAM-B3LYP is important for a better characterization of the aromaticity since they present much smaller delocalization errors than B3LYP. Given these considerations and the fact that the errors associated with CAM-B3LYP are deemed acceptable, the present study will use the CAM-B3LYP/cc-pVTZ level of theory.

S1.2. Indicators of aromaticity

A fundamental characteristic of aromatic compounds is their cyclic electron delocalization. Consequently, the evaluation of aromaticity can be performed by quantifying this electron delocalization. Definitions such as higher-order electron sharing indices, obtained specifically from QTAIM (quantum theory of atoms in molecules) space partitioning, have been widely tested and proven to give accurate aromaticity descriptions[35-37]. Additionally, the electron density of delocalized bonds (EDDB)[30-32], based on Hilbert partitioning, effectively accounts for multicenter bonding at a reasonable cost.

However, the accuracy of individual aromaticity measures cannot be determined since aromaticity cannot be directly measured by any physical or chemical experiment. This limits the establishment of unambiguous reference values, making it necessary to use a variety of indices. Consistent results across multiple indices strengthen confidence in the assessment of aromaticity. Understanding the specific characteristics of each aromaticity index is crucial for clearer interpretations and identifying inconsistencies. This section defines and describes the main features of the electronic indices used in this work.

Assessing aromaticity involves comparing the values obtained for the molecule under study with a benchmark aromatic compound, typically benzene. For instance, the fluctuation index (FLU)[24,25] compares the delocalization indices (DI) of atom pairs in a ring ($\mathcal{A} = A_1, A_2, \ldots, A_n$) to those of well-known aromatic molecules (*e.g.*, benzene for C−C, and pyridine or pyrrole for C−N bonds):

$$FLU = \frac{1}{n}\sum_{i=1}^{n}\left[\left(\frac{\delta(A_i)}{\delta(A_{i-1})}\right)^{\alpha}\left(\frac{\delta(A_i,A_{i-1})-\delta_{ref}(A_i,A_{i-1})}{\delta_{ref}(A_i,A_{i-1})}\right)\right]^2, \qquad (2)$$

where α is defined as:

$$\alpha = \begin{cases} 1\ if\ \delta(A_{i-1}) \leq \delta(A_i) \\ -1\ if\ \delta(A_i) < \delta(A_{i-1}) \end{cases} \quad (3)$$

In this equation, δ(A) is the atomic valence for a closed-shell system, and the α function ensures values greater than or equal to one. According to FLU, aromatic molecules present small values, close to zero (exactly zero for benzene), and larger values for non-aromatic or antiaromatic molecules. Indices based on references are more suited for evaluating the relative aromaticity of compounds resembling their references, like substituted benzenes, small annulenes, or small heteroaromatics. However, they may fail in cases like the transition state of the Diels-Alder reaction,[36, 38] or the identification of the annulene pathway as the most aromatic in 18H porphyrin[20]. FLU can only be used for molecular systems with available DI reference values, excluding metal aromaticity, systems with halogens, or bonds deviating from those typically found in classical organic molecules. This limitation might be addressed by establishing DI values for aromaticity in these systems.

The preferred approach to improve aromaticity evaluation is to consider electron delocalization among multiple atoms simultaneously. The $I_{ring}(\mathcal{A})$[29] and MCI[28] indices, which measure electron delocalization over all atoms in a ring:

$$I_{ring}(\mathcal{A}) = \sum_{i_1, i_2, \ldots, i_n} n_{i_1}, \ldots, n_{i_n} S_{i_1 i_2}(A_1)\, S_{i_2 i_3}(A_2) \ldots S_{i_n i_1}(A_n), \quad (4)$$

where n_i is the orbital occupancy and $S_{ij}(A_n)$ is the overlap of molecular spinorbitals in the atom A_n. The I_{ring} values for reference aromatic (benzene), non-aromatic (cyclohexane), and antiaromatic (cyclohexatriene with a ratio of 0.8 between two consecutive bond distances (*a*/*b*)) molecules are 0.048, 0.000, and 0.012, respectively, as calculated using the CAM-B3LYP/6-311G(d,p) level of theory[37].

The multicenter index MCI($\mathcal{A}$)[28] measures electron delocalization by averaging overlap integrals over all permutations of the *n* atoms in $\mathcal{A}$:

$$MCI(\mathcal{A}) = \frac{1}{2n} \sum_{\mathcal{P}(\mathcal{A})} I_{ring}(\mathcal{A}), \quad (5)$$

were $\mathcal{P}(\mathcal{A})$ represents the *n*! permutations of the elements in $\mathcal{A}$. Thus, it considers not only the Kekulé but all structures resulting from taking into account all possible bonding arrangements between the atoms in the ring. Using MCI aromatic compounds provide large positive numbers, and small or even negative numbers are obtained for non-aromatic and antiaromatic species. For instance, the MCI (normalized MCI: $MCI^{1/n}$) CAM-B3LYP/6-311G(d,p) values are 0.072 (0.646), 0.000 (0.260), and 0.013 (0.484) for benzene, cyclohexane, and cyclohexatriene (*a/b* = 0.8), respectively[37].

An important limitation of multicenter approaches, especially MCI, is that they are only feasible options for rings of small to medium size, with up to 14 members[26]. With the aim to overcome these drawbacks, in 2016 Matito proposed the AV1245 index26 based on the simultaneous delocalization among four atom fragments. This allows for the evaluation of extended delocalization while maintaining a reasonable computational cost. AV1245 is calculated as the arithmetic mean of the

successive four-center multicenter index (4c-MCI) for the relative atomic positions 1−2 and 4−5 along the perimeter of the ring.

Examining the results of the AV1245 index, the same as for previously discussed indices, the higher the value, the more aromatic the compound is. For benzene, cyclohexane, and cyclohexatriene (a/b = 0.8) the AV1245 CAM-B3LYP/6-311G(d,p) value is 10.72, -0.01, and 2.99, respectively[37]. This index has been specifically created for analyzing large rings, thus AV1245 cannot be calculated for rings of less than six members. Notably, the AV1245 index values tend to decrease as the size of the system increases, regardless of its aromatic nature. The AV1245 values at CAM-B3LYP/6-311G(d,p) level of theory for aromatic 18H porphyrin and $C_{18}H_{18}$ are 2.16[20] and 1.80, respectively,[39] which are significantly low compared to the value of benzene. A decrease in the aromaticity values in systems with larger n is inherent to the index's calculation. Mathematically, indices based on n-center-MCIs involve the product of terms, each of which is a number less than unity. As the ring size increases, the number of these multiplicative terms rises, leading to a cumulative effect where the overall product becomes progressively smaller.

The last method based on electronic criteria we review is the electron density of delocalized bonds (EDDB) [30-32]. It is based on decomposing the one-electron density (ED) into different terms corresponding to the electron density localized on atoms (EDLAs) that includes the core electrons, lone pairs, and so on, the electron density of localized bonds (EDLBs) representing the Lewis-like 2-center 2-electron bonds, and the electron density of delocalized bonds which includes the rest of the density that cannot be assigned to atoms or bonds due to its delocalized nature. The latter term accounts for the conjugation of a bond with the rest of 2c and 3c bonds in the molecule.

$$\mathrm{ED}(\mathbf{r}) = \mathrm{EDLA}(\mathbf{r}) + \mathrm{EDLB}(\mathbf{r}) + \mathrm{EDDB}(\mathbf{r}) \tag{6}$$

S2. UV-Vis Spectra and Frontier Molecular Orbitals

S2.1 Experimental UV-Vis absorption spectra

In the following figures, we present the experimental results derived from Ultraviolet-Visible (UV-Vis) spectroscopy for a **SubPz** derivative, **SubPc**, **Pz**, and **Pc** conducted in Tetrahydrofuran (THF) and the comparative of experimental and theoretical results.

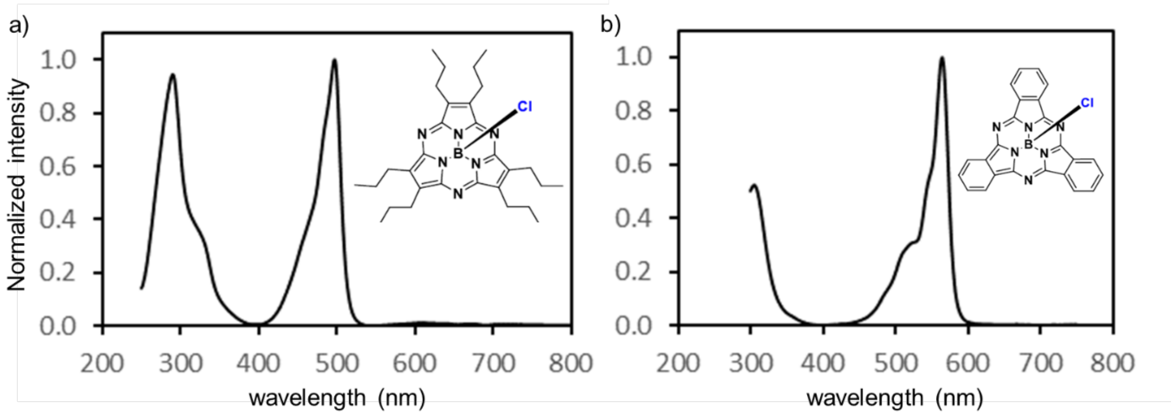

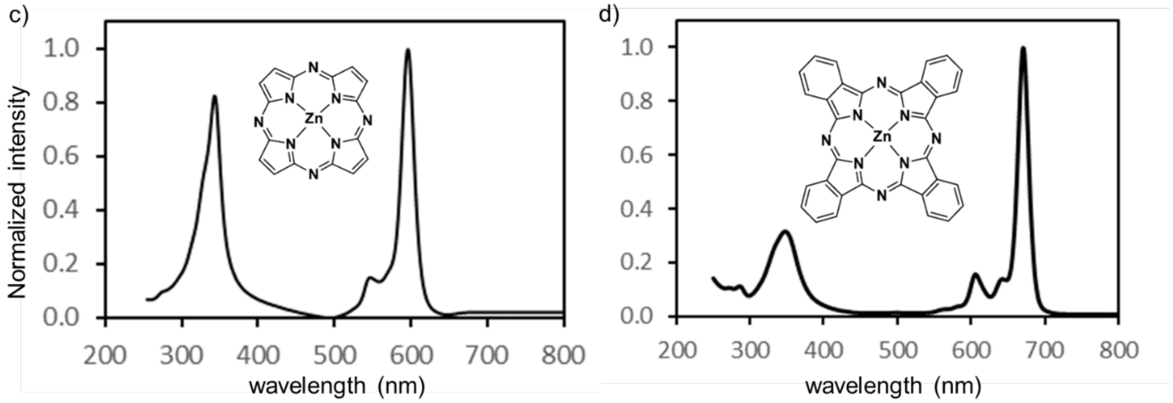

Figure S2. Absorption spectra of a) **SubPz** derivative, b) **SubPc**, c) **Pz**, and d) **Pc** in THF.

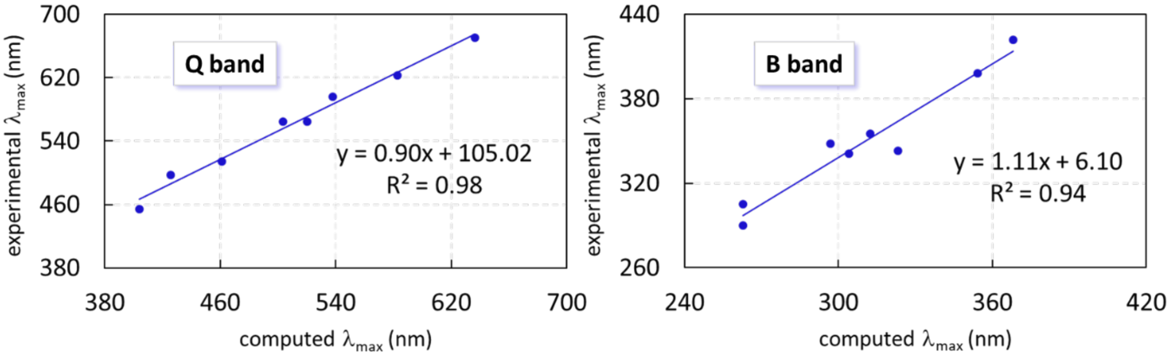

Figure S3. Linear Correlation of Experimental and Theoretical Absorption Maxima.

S2.2 Computational UV-Vis absorption spectra and molecular orbitals

The following tables present the detailed absorption spectra results computed with CAM-B3LYP using a cc-pVTZ basis set and PCM to account for the effect of the corresponding solvent for the nine studied systems. The Q and B bands are represented by a pair of degenerate excited states, resulting from the D_{4h} and C_{3v} symmetries of the tetra- or tri-pyrrole/isoindole systems. To streamline our discussion, we will refer to the set of **P**, **Pz**, **TBP**, and **Pc** as phthalocyanines and **ZnSubP**, **SubP**, **SubPz**, **TBSubP**, and **SubPc** as subphthalocyanines. In all cases, the Q band arises from the first two singlet excited states (H-1, H → L, L+1 transitions), while the B band originates from higher-lying singlet states (generally involving the same orbitals). The latter states correspond to S_3 and S_4 in systems having CH at the *meso* position, or higher singlets for systems containing N–*meso*. This outcome was expected, given that the a_{2u} (H-1 in phthalocyanines) and a_1 (H-1 in subphthalocyanines) orbitals (see Figure 1 in the manuscript and Table S17), are significantly stabilized in the presence of N–*meso*. In the same vein, we could also anticipate the difference in the oscillator strength (*f*) between systems with CH or N *meso*-substitution. **P**, **TBP**, **ZnSubP**, **SubP**, and **TBSubPc** have lower *f* values

than their N–*meso* counterparts for the Q band, while the situation is reversed for the B band (see Table 1 in the manuscript and Tables S8 to S16; for instance, compare the results of Tables S13 and S14).

The contraction from phthalocyanines and subphthalocyanines results into larger H–L gaps, compared to phthalocyanines, that give rise to the blue shifting of absorption Q bands. Similarly, B bands are also blue shifted upon contraction. The substituted phthalocyanines (**Pz**, **TBP**, and **Pc)** exhibit red and blue shifts in the Q and B bands, respectively, compared to **P**. While the band position is shifted, the intensities (quantified by *f*) of Q and B bands in **Pc** and **SubPc** are also affected. With respect to **P**, the Q band in **Pc** and **SubPc** exhibits an increased intensity, while the B band shows a decrease. However, these variations in intensity, as determined by our computational analysis, do not alter the order of the intensities of these bands, as experimentally found.[40,41]

In comparison to **P** reference, **SubP**, **SubPz**, and **TBSubP** show a blue shift in both Q and B bands in agreement with the increase of the spacing between H and L orbitals in these contracted systems (Figure 2 in the manuscript). In the case of **SubPc**, the Q band is observed at wavelengths comparable to those in **P**, while the predicted H-L energy gap is 0.2 eV narrower in **SubPc**. For the B band of this system, a blue shift is observed, consistent with the trend seen in the other subphthalocyanines.

Finally, the comparison between **SubP** and **ZnSubP** confirms that the previously mentioned changes are not due to the central coordination, thereby primarily attributing the blue shifts with respect to **P** to adjustments within the π-system and other differences in the carbon-nitrogen framework.

Table S8. TDDFT calculations for the **P** system, computed at CAM-B3LYP/cc-pVTZ level of theory using EtOH as implicit solvent. Orbital symmetry labels for the most relevant transitions corresponding to Q and B bands are indicated in parentheses.

root	symm.	transition	contribution	energy (eV)	wavelength (nm)	*f*
1	E_u	$94(a_{2u}) \rightarrow 97(e_g)$<br>$95(a_{1u}) \rightarrow 96(e_g)$	-0.48<br>0.52	2.4	520.3	0.010
2	E_u	$94(a_{2u}) \rightarrow 96(e_g)$<br>$95(a_{1u}) \rightarrow 97(e_g)$	0.48<br>0.52	2.4	520.3	0.010
3	E_u	$94(a_{2u}) \rightarrow 96(e_g)$<br>$95(a_{1u}) \rightarrow 97(e_g)$	0.52<br>-0.48	3.5	354.1	1.370
4	E_u	$94(a_{2u}) \rightarrow 97(e_g)$<br>$95(a_{1u}) \rightarrow 96(e_g)$	0.52<br>0.48	3.5	354.1	1.370
5	E_g	$93 \rightarrow 96$<br>$93 \rightarrow 97$	0.57<br>0.41	3.8	325.3	0.000
6	E_g	$93 \rightarrow 96$<br>$93 \rightarrow 97$	-0.41<br>0.57	3.8	325.3	0.000
7	–	$90 \rightarrow 97$<br>$91 \rightarrow 96$	-0.48<br>0.48<br>0.16	4.3	291.7	0.000

		92 → 98				
8	–	89 → 98<br>90 → 97<br>91 → 96	0.15<br>0.48<br>0.48	4.3	287.5	0.000
9	E_u	89 → 96<br>91 → 98<br>92 → 96	0.21<br>0.16<br>0.65	4.4	280.1	0.053
10	E_u	89 → 97<br>90 → 98<br>92 → 97	-0.21<br>-0.16<br>0.65	4.4	280.1	0.053

Table S9. TDDFT calculations for the **Pz** system, computed at CAM-B3LYP/cc-pVTZ level of theory using THF as implicit solvent. Orbital symmetry labels for the most relevant transitions corresponding to Q and B bands are indicated in parentheses.

root	Symm.	transition	contribution	energy (eV)	wavelength (nm)	*f*
1	E_u	93(a_{2u}) → 97(e_g)<br>95(a_{1u}) → 96(e_g)	0.24<br>0.67	2.3	537.9	0.316
2	E_u	93(a_{2u}) → 96(e_g)<br>95(a_{1u}) → 97(e_g)	-0.24<br>0.67	2.3	537.9	0.316
3	E_g	94 → 97	0.70	3.1	405.0	0.000
4	E_g	94 → 96	0.70	3.1	405.0	0.000
5	–	90 → 96<br>91 → 97<br>92 → 98	-0.48<br>0.48<br>-0.18	3.8	327.4	0.000
6	E_u	88 → 97(e_g)<br>91 → 98<br>92 → 97(e_g)<br>93(a_{2u}) → 97(e_g)<br>95(a_{1u}) → 96(e_g)	0.27<br>-0.16<br>0.49<br>0.38<br>-0.16	3.8	323.2	0.226
7	E_u	88 → 96(e_g)<br>90 → 98<br>92 → 96(e_g)<br>93(a_{2u}) → 96(e_g)<br>95(a_{1u}) → 97(e_g)	-0.27<br>0.16<br>0.49<br>-0.38<br>-0.16	3.8	323.2	0.226
8	–	88 → 98<br>90 → 96<br>91 → 97	-0.15<br>0.49<br>0.49	3.8	322.2	0.000
9	E_g	87 → 98<br>89 → 96	0.16<br>0.68	3.9	316.2	0.000
10	E_g	86 → 98<br>89 → 97	0.16<br>0.68	3.9	316.2	0.000

Table S10. TDDFT calculations for the **TBP** system computed at CAM-B3LYP/cc-pVTZ level of theory using EtOH as implicit solvent. Orbital symmetry labels for the most relevant transitions corresponding to Q and B bands are indicated in parentheses.

root	Symm.	transition	contribution	energy (eV)	wavelength (nm)	f
1	E_u	$146(a_{2u}) \rightarrow 149(e_g)$<br>$147(a_{1u}) \rightarrow 148(e_g)$	-0.30<br>0.64	2.1	582.9	0.304
2	E_u	$146(a_{2u}) \rightarrow 148(e_g)$<br>$147(a_{1u}) \rightarrow 149(e_g)$	0.30<br>0.64	2.1	582.9	0.304
3	E_u	$146(a_{2u}) \rightarrow 148(e_g)$<br>$147(a_{1u}) \rightarrow 149(e_g)$<br>$147(a_{1u}) \leftarrow 149(e_g)$	0.63<br>-0.31<br>0.10	3.4	368.1	1.603
4	E_u	$146(a_{2u}) \rightarrow 149(e_g)$<br>$147(a_{1u}) \rightarrow 148(e_g)$<br>$147(a_{1u}) \leftarrow 148(e_g)$	0.63<br>0.31<br>-0.10	3.4	368.1	1.603
5	B_{2g}	$144 \rightarrow 148$<br>$145 \rightarrow 149$<br>$147 \rightarrow 150$	-0.13<br>-0.13<br>0.65	4.1	302.3	0.000
6	B_{1g}	$144 \rightarrow 149$<br>$145 \rightarrow 148$<br>$147 \rightarrow 152$	-0.21<br>0.21<br>0.62	4.1	301.4	0.000
7	E_g	$140 \rightarrow 148$<br>$140 \rightarrow 149$	-0.13<br>0.69	4.1	299.9	0.000
8	E_g	$140 \rightarrow 148$<br>$140 \rightarrow 149$	0.69<br>0.13	4.1	299.9	0.000
9	A_{2g}	$144 \rightarrow 148$<br>$145 \rightarrow 149$<br>$147 \rightarrow 151$	0.12<br>-0.12<br>0.66	4.2	298.7	0.000
10	–	$141 \rightarrow 148$<br>$142 \rightarrow 149$<br>$144 \rightarrow 148$<br>$145 \rightarrow 149$<br>$147 \rightarrow 151$	0.14<br>-0.14<br>-0.44<br>0.44<br>0.21	4.3	287.1	0.000

Table S11. TDDFT calculations for the **Pc** system computed at CAM-B3LYP/cc-pVTZ level of theory using THF as implicit solvent. Orbital symmetry labels for the most relevant transitions corresponding to Q and B bands are indicated in parentheses.

root	Symm.	transition	contribution	energy (eV)	wavelength (nm)	f
1	E_u	$146(a_{2u}) \rightarrow 149(e_g)$<br>$147(a_{1u}) \rightarrow 148(e_g)$	0.14<br>0.69	1.9	636.3	0.675
2	E_u	$146(a_{2u}) \rightarrow 148(e_g)$<br>$147(a_{1u}) \rightarrow 149(e_g)$	-0.14<br>0.69	1.9	636.3	0.675
3	E_g	$142 \rightarrow 148$	0.70	3.5	353.1	0.000
4	E_g	$142 \rightarrow 149$	0.70	3.5	353.1	0.000
5	B_{1g}	$143 \rightarrow 148$<br>$144 \rightarrow 149$<br>$147 \rightarrow 150$	-0.12<br>-0.12<br>0.66	3.9	320.2	0.000

6	–	143 → 149<br>144 → 148<br>145 → 150	0.47<br>0.47<br>0.16	3.9	318.1	0.000
7	–	138 → 150<br>143 → 149<br>144 → 148<br>147 → 151	-0.13<br>-0.47<br>0.47<br>0.16	3.9	316.8	0.000
8	E_u	138 → 148<br>144 → 150<br>145 → 148	-0.36<br>0.15<br>0.56	4.0	312.6	0.097
9	E_u	138 → 149<br>143 → 150<br>145 → 149	0.36<br>0.15<br>0.56	4.0	312.6	0.097
10	E_g	135 → 150<br>137 → 148<br>137 → 149	0.12<br>0.11<br>0.67	4.2	297.9	0.000
11	E_g	134 → 150<br>137 → 148<br>137 → 149<br>136 → 148	-0.12<br>0.67<br>-0.11<br>0.10	4.2	297.9	0.000
12	E_u	138 → 149(e_g)<br>139 → 148(e_g)<br>145 → 149(e_g)<br>146(a_{2u}) → 149(e_g)<br>147(a_{1u}) → 148(e_g)<br>136 → 149(e_g)	-0.18<br>-0.14<br>0.20<br>0.60<br>-0.14<br>-0.10	4.2	297.0	1.139
13	E_u	138 → 148(e_g)<br>139 → 149(e_g)<br>145 → 148(e_g)<br>146(a_{2u}) → 148(e_g)<br>147(a_{1u}) → 149(e_g)<br>134 → 150	-0.18<br>-0.14<br>-0.20<br>0.60<br>0.14<br>-0.12	4.2	297.0	1.139

Table S12. TDDFT calculations for the **ZnSubP** system computed at CAM-B3LYP/cc-pVTZ level of theory using DCM as implicit solvent. Orbital symmetry labels for the most relevant transitions corresponding to Q and B bands are indicated in parentheses.

root	Symm.	transition	contribution	energy (eV)	wavelength (nm)	*f*
1	E	74(a_1) → 77(e)<br>75(a_2) → 76(e)	0.41<br>0.57	3.0	415.4	0.028
2	E	74(a_1) → 76(e)<br>75(a_2) → 77(e)	-0.41<br>0.57	3.0	415.4	0.028
3	E	74(a_1) → 77(e)<br>75(a_2) → 76(e)	0.56<br>-0.41	4.0	308.4	0.887
4	E	74(a_1) → 76(e)<br>75(a_2) → 77(e)	0.56<br>0.41	4.0	308.4	0.887
5	A_2	75 → 78	0.70	4.1	302.1	0.000

6	A_1	74 → 78	0.70	4.2	294.5	0.013
7	–	72 → 76<br>73 → 77	-0.49<br>0.49	4.3	290.4	0.000
8	–	71 → 77<br>72 → 76<br>73 → 77	0.17<br>0.47<br>0.47	4.5	275.7	0.033
9	–	71 → 76<br>72 → 77<br>73 → 76	-0.17<br>-0.47<br>0.47	4.5	275.7	0.033
10	–	72 → 77<br>73 → 76	0.48<br>0.48	4.9	253.6	0.017

Table S13. TDDFT calculations for the **SubP** system computed at CAM-B3LYP/cc-pVTZ level of theory using DCM as implicit solvent. Orbital symmetry labels for the most relevant transitions corresponding to Q and B bands are indicated in parentheses.

root	Symm.	transition	contribution	energy (eV)	wavelength (nm)	*f*
1	*E*	70(a_1) → 72(e)<br>71(a_2) → 73(e)	-0.43<br>0.56	3.1	403.9	0.025
2	*E*	70(a_1) → 73(e)<br>71(a_2) → 72(e)	0.43<br>0.56	3.1	403.9	0.025
3	*E*	67 → 72(e)<br>70(a_1) → 72(e)<br>71(a_2) → 73(e)	-0.11<br>0.55<br>0.43	4.1	304.2	0.855
4	*E*	67 → 73(e)<br>70(a_1) → 73(e)<br>71(a_2) → 72(e)	-0.11<br>0.55<br>-0.43	4.1	304.1	0.854
5	–	68 → 72<br>69 → 73	-0.49<br>0.49	4.5	276.0	0.000
6	–	67 → 73<br>68 → 72<br>69 → 73	0.24<br>0.46<br>0.46	4.7	264.7	0.037
7	–	67 → 72<br>68 → 73<br>69 → 72	-0.24<br>-0.46<br>0.46	4.7	264.7	0.037
8	–	68 → 73<br>69 → 72	0.49<br>0.49	5.1	241.1	0.016
9	*E*	67 → 72<br>68 → 73<br>69 → 72	0.65<br>-0.17<br>0.17	5.4	227.9	0.184
10	*E*	67 → 73<br>68 → 72<br>69 → 73	0.65<br>-0.17<br>-0.17	5.4	227.9	0.184

Table S14. TDDFT calculations for the **SubPz** system computed at CAM-B3LYP/cc-pVTZ level of theory using THF as implicit solvent. Orbital symmetry labels for the most relevant transitions corresponding to Q and B bands are indicated in parentheses.

root	Symm.	transition	contribution	energy (eV)	wavelength (nm)	f
1	E	$70(a_1) \rightarrow 73(e)$<br>$71(a_2) \rightarrow 72(e)$	0.20<br>0.68	2.9	425.7	0.221
2	E	$70(a_1) \rightarrow 72(e)$<br>$71(a_2) \rightarrow 73(e)$	-0.20<br>0.68	2.9	425.7	0.221
3	–	$68 \rightarrow 72$<br>$69 \rightarrow 73$	0.49<br>-0.49	4.1	305.3	0.000
4	–	$67 \rightarrow 73$<br>$68 \rightarrow 73$<br>$69 \rightarrow 72$	0.28<br>-0.45<br>0.45	4.3	290.6	0.000
5	–	$67 \rightarrow 72$<br>$68 \rightarrow 72$<br>$69 \rightarrow 73$	0.28<br>0.45<br>0.45	4.3	290.6	0.000
6	–	$63 \rightarrow 72$<br>$64 \rightarrow 73$<br>$65 \rightarrow 72$<br>$66 \rightarrow 73$	0.13<br>-0.13<br>-0.48<br>0.48	4.4	281.6	0.002
7	–	$62 \rightarrow 73$<br>$65 \rightarrow 72$<br>$66 \rightarrow 73$<br>$67 \rightarrow 73$	0.17<br>0.45<br>0.45<br>0.16	4.6	269.1	0.006
8	–	$62 \rightarrow 72$<br>$65 \rightarrow 73$<br>$66 \rightarrow 72$<br>$67 \rightarrow 72$	-0.17<br>-0.45<br>0.45<br>-0.16	4.6	269.1	0.006
9	E	$67 \rightarrow 72(e)$<br>$70(a_1) \rightarrow 72(e)$<br>$71(a_2) \rightarrow 73(e)$	-0.22<br>0.62<br>0.18	4.7	262.7	0.458
10	E	$67 \rightarrow 73(e)$<br>$70(a_1) \rightarrow 73(e)$<br>$71(a_2) \rightarrow 72(e)$	-0.22<br>0.62<br>-0.18	4.7	262.7	0.459

Table S15. TDDFT calculations for the **TBSubP** system computed at CAM-B3LYP/cc-pVTZ level of theory using DCM as implicit solvent. Orbital symmetry labels for the most relevant transitions corresponding to Q and B bands are indicated in parentheses.

root	Symm.	transition	contribution	energy (eV)	wavelength (nm)	f
1	E	$109(a_1) \rightarrow 111(e)$<br>$110(a_2) \rightarrow 112(e)$	-0.24<br>0.66	2.7	461.0	0.308
2	E	$109(a_1) \rightarrow 112(e)$<br>$110(a_2) \rightarrow 111(e)$	0.24<br>0.66	2.7	461.0	0.308
3	E	$109(a_1) \rightarrow 111(e)$<br>$110(a_2) \rightarrow 112(e)$	0.65<br>0.25	4.0	312.4	1.055
4	E	$109(a_1) \rightarrow 112(e)$<br>$110(a_2) \rightarrow 111(e)$	0.65<br>-0.25	4.0	312.4	1.055
5	E	$107 \rightarrow 111$	-0.18	4.4	284.0	0.047

		108 → 112<br>110 → 114	0.18<br>0.63			
6	*E*	107 → 112<br>108 → 111<br>110 → 115	0.18<br>0.18<br>0.63	4.4	284.0	0.048
7	A_2	107 → 112<br>108 → 111<br>110 → 113	-0.15<br>0.14<br>0.65	4.4	283.4	0.000
8	–	107 → 112<br>108 → 111<br>110 → 113	-0.46<br>0.46<br>-0.23	4.6	267.3	0.000
9	–	104 → 111<br>107 → 111<br>108 → 112<br>110 → 114	0.21<br>-0.42<br>0.42<br>-0.28	4.8	260.8	0.003
10	–	104 → 112<br>107 → 112<br>108 → 111<br>110 → 115	0.21<br>0.42<br>0.42<br>-0.28	4.8	260.8	0.003

Table S16. TDDFT calculations for the **SubPc** system computed at CAM-B3LYP/cc-pVTZ level of theory using THF as implicit solvent. Orbital symmetry labels for the most relevant transitions corresponding to Q and B bands are indicated in parentheses.

root	Symm.	transition	contribution	energy (eV)	wavelength (nm)	*f*
1	*E*	107(a_1) → 112(e)<br>110(a_2) → 111(e)	-0.10<br>0.69	2.5	503.3	0.475
2	*E*	107(a_1) → 111(e)<br>110(a_2) → 112(e)	0.10<br>0.69	2.5	503.3	0.475
3	–	108 → 111<br>109 → 112<br>110 → 115	-0.50<br>0.47<br>-0.20	4.1	299.2	0
4	–	104 → 112<br>107 → 112<br>108 → 112<br>109 → 111<br>110 → 114	-0.10<br>-0.20<br>0.38<br>-0.40<br>-0.40	4.2	293.2	0.026
5	–	104 → 111<br>107 → 111<br>108 → 111<br>109 → 112<br>110 → 113	0.13<br>0.19<br>0.38<br>0.38<br>0.35	4.2	293.2	0.026
6	–	108 → 111<br>109 → 112<br>110 → 115	-0.10<br>0.11<br>0.68	4.4	278.7	0
7	–	104 → 111<br>107 → 111	-0.20<br>-0.10	4.5	277.7	0.036

		108 → 111	-0.20			
		109 → 112	-0.20			
		110 → 113	0.59			
		104 → 112	-0.20			
		107 → 112	-0.10			
8	–	108 → 112	0.22	4.5	277.7	0.036
		109 → 111	-0.20			
		110 → 114	0.59			
		101 → 111	-0.10			
		102 → 112	-0.10			
		105 → 111	-0.40			
		106 → 112	0.37			
9	–	107 → 115	-0.10	4.6	270.5	0.001
		108 → 112	-0.30			
		108 → 113	-0.10			
		109 → 111	-0.30			
		109 → 114	0.13			
		104 → 111(e)	-0.15			
		107(a_1) → 111(e)	0.62			
10	*E*	108 → 111(e)	-0.12	4.7	262.9	0.866
		109 → 112(e)	-0.12			
		110(a_2) → 112(e)	-0.12			
		104 → 112(e)	-0.15			
		107(a_1) → 112(e)	0.62			
11	*E*	108 → 112(e)	0.12	4.7	262.9	0.866
		109 → 111(e)	-0.12			
		110(a_2) → 111(e)	0.12			

To draw comparisons with available experimental results, we computed the UV-Vis absorption spectra in a solvent environment. However, our focus lies in studying the orbital energy gaps in the gas phase. The rationale behind this methodology is to reduce the cost of the calculations as much as possible, thereby allowing the use of gas-phase gaps as straightforward predictive tools. To confirm that the solvation does not have a major effect, we compared the results obtained in both the solvent and gas phase. As illustrated in Table S19, the solvent has a minimal effect on the calculated orbital energy differences (the differences in $\Delta\varepsilon$_a_{1u}-e_g and $\Delta\varepsilon$_a_{2u}-e_g (or $\Delta\varepsilon$_a_2-e and $\Delta\varepsilon$_a_1-e) are less than 0.1 eV in all cases). The only factor to consider is the order of the molecular orbitals, which can be altered upon inclusion of solvent effects (see Table S18).

Table S17. The six frontier molecular orbitals (HOMO-3, HOMO-2, HOMO-1, HOMO, LUMO, and LUMO+1) for **P**, **TBP**, **Pz**, **Pc**, **Zn-SubP**, **SubP**, **TBSubP**, **SubPz**, and **SubPc** systems with CAM-B3LYP/cc-pVTZ. The isocontour is 0.02 a.u. and the orbital energies are in eV.

	**HOMO-3**	**HOMO-2**	**HOMO-1**	**HOMO**	**LUMO**	**LUMO+1**
**P**	MO 92<br>b_{2u}<br>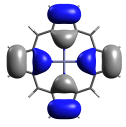<br>-8.431	MO 93<br>b_{1g}<br>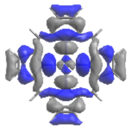<br>-8.268	MO 94<br>a_{2u}<br>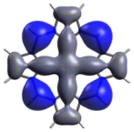<br>-6.515	MO 95<br>a_{1u}<br>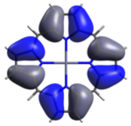<br>-6.379	MO 96<br>e_g<br>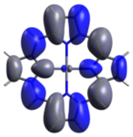<br>-1.562	MO 97<br>e_g<br>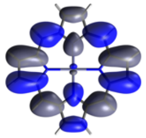<br>-1.562
**Pz**	MO 92<br>b_{2u}<br>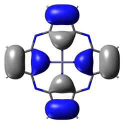<br>-8.957	MO 93<br>b_{1g}<br>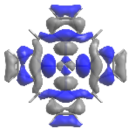<br>-8.501	MO 94<br>a_{2u}<br>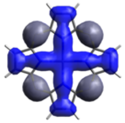<br>-8.458	MO 95<br>a_{1u}<br>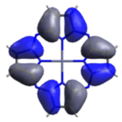<br>-6.762	MO 96<br>e_g<br>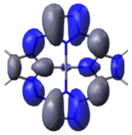<br>-2.431	MO 97<br>e_g<br>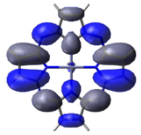<br>-2.431
**TBP**	MO 144<br>e_g<br>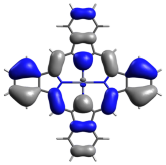<br>-8.096	MO 145<br>e_g<br>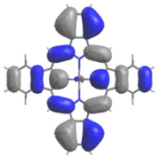<br>-8.096	MO 146<br>a_{2u}<br>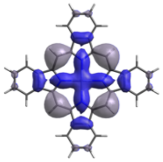<br>-6.702	MO 147<br>a_{1u}<br>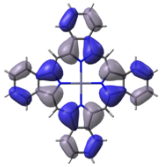<br>-5.678	MO 148<br>e_g<br>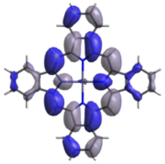<br>-1.547	MO 149<br>e_g<br>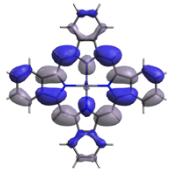<br>-1.547
**Pc**	MO 144<br>e_g<br>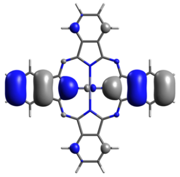<br>-8.455	MO 145<br>b_{2u}<br>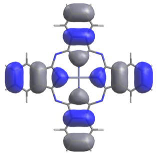<br>-8.409	MO 146<br>a_{2u}<br>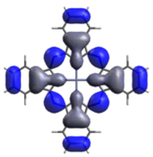<br>-8.352	MO 147<br>a_{1u}<br>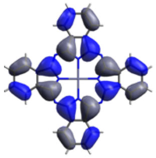<br>-5.889	MO 148<br>e_g<br>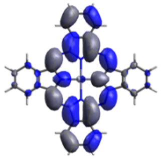<br>-2.163	MO 149<br>e_g<br>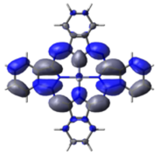<br>-2.163

**ZnSubP**	MO 72<br>*e*<br>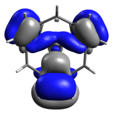<br>-8.133	MO 73<br>*e*<br>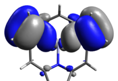<br>-8.133	MO 74<br>a_1<br>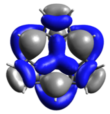<br>-6.670	MO 75<br>a_2<br>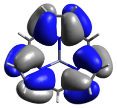<br>-6.357	MO 76<br>a_1<br>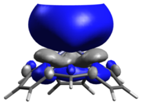<br>-0.980	MO 77<br>*e*<br>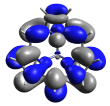<br>-0.736
**SubP**	MO 68<br>*e*<br>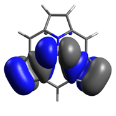<br>-8.577	MO 69<br>*e*<br>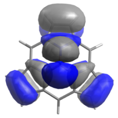<br>-8.577	MO 70<br>a_1<br>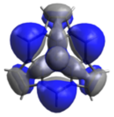<br>-7.208	MO 71<br>a_2<br>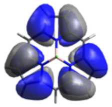<br>-7.009	MO 72<br>*e*<br>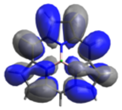<br>-1.322	MO 73<br>*e*<br>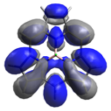<br>-1.322
**SubPz**	MO 68<br>*e*<br>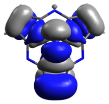<br>-9.19	MO 69<br>*e*<br>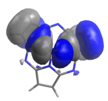<br>-9.19	MO 70<br>a_1<br>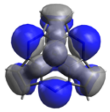<br>-9.06	MO 71<br>a_2<br>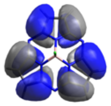<br>-7.39	MO 72<br>*e*<br>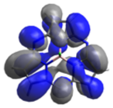<br>-2.13	MO 73<br>*e*<br>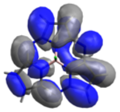<br>-2.13
**TBSubP**	MO 107<br>*e*<br>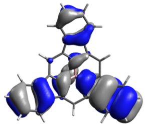<br>-8.335	MO 108<br>*e*<br>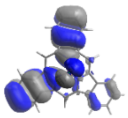<br>-8.335	MO 109<br>a_1<br>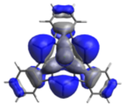<br>-7.282	MO 110<br>a_2<br>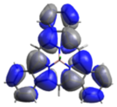<br>-6.168	MO 111<br>*e*<br>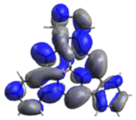<br>-1.252	MO 112<br>*e*<br>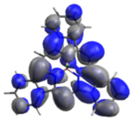<br>-1.252
**SubPc**	MO 107<br>a_1<br>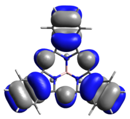<br>-8.71	MO 108<br>*e*<br>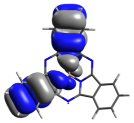<br>-8.64	MO 109<br>*e*<br>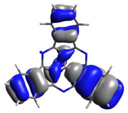<br>-8.64	MO 110<br>a_2<br>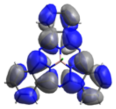<br>-6.409	MO 111<br>*e*<br>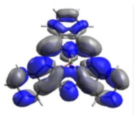<br>-1.842	MO 112<br>*e*<br>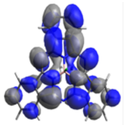<br>-1.842

Table S18. Changes in order of the molecular orbitals in a) D_{4h} and b) C_{3v} symmetry systems at CAM-B3LYP/cc-pVTZ level of theory with and without considering an implicit solvent (scrf=(pcm,solvent)). See experimental data section in the manuscript for the solvent used in each case.

a)	Molecular Orbital		CAM-B3LYP gas	CAM-B3LYP solv.
**P**	H-3	92	b_{2u}	b_{2u}
	H-2	93	b_{1g}	b_{1g}
	H-1	94	a_{2u}	a_{2u}
	H	95	a_{1u}	a_{1u}
	L	96	e_g	e_g
	L+1	97	e_g	e_g
**Pz**	H-3	92	b_{2u}	b_{2u}
	H-2	93	b_{1g}	a_{2u}
	H-1	94	a_{2u}	b_{1g}
	H	95	a_{1u}	a_{1u}
	L	96	e_g	e_g
	L+1	97	e_g	e_g
**TBP**	H-3	144	e_g	e_g
	H-2	145	e_g	e_g
	H-1	146	a_{2u}	a_{2u}
	H	147	a_{1u}	a_{1u}
	L	148	e_g	e_g
	L+1	149	e_g	e_g
**Pc**	H-3	144	e_g	e_g
	H-2	145	b_{2u}	b_{2u}
	H-1	146	a_{2u}	a_{2u}
	H	147	a_{1u}	a_{1u}
	L	148	e_g	e_g
	L+1	149	e_g	e_g

b)	Molecular Orbital		CAM-B3LYP gas	CAM-B3LYP solv.
**ZnSubP**	H-3	72	e	e
	H-2	73	e	e
	H-1	74	a_1	a_1
	H	75	a_2	a_2
	L	76	a_1	e
	L+1	77	e	e
**SubP**	H-3	68	e	e
	H-2	69	e	e
	H-1	70	a_1	a_1
	H	71	a_2	a_2
	L	72	e	e
	L+1	73	e	e
**SubPz**	H-3	68	e	e
	H-2	69	e	e
	H-1	70	a_1	a_1
	H	71	a_2	a_2
	L	72	e	e
	L+1	73	e	e
**TBSubP**	H-3	107	e	e

	H-2	108	*e*	*e*
	H-1	109	a_1	a_1
	H	110	a_2	a_2
	L	111	*e*	*e*
	L+1	112	*e*	*e*
**SubPc**	H-3	107	a_1	a_1
	H-2	108	*e*	*e*
	H-1	109	*e*	*e*
	H	110	a_2	a_2
	L	111	*e*	*e*
	L+1	112	*e*	*e*

Table S19. Summary of the $\Delta\varepsilon\_a_{1u}\text{-}e_g$ and $\Delta\varepsilon\_a_{2u}\text{-}e_g$ (or $\Delta\varepsilon\_a_2\text{-}e$ and $\Delta\varepsilon\_a_1\text{-}e$) in eV at CAM-B3LYP/cc-pVTZ level of theory with and without considering an implicit solvent (scrf=(pcm,solvent)). See experimental data section in the manuscript for the solvent used in each case.

	$\Delta\varepsilon\_a_{1u}\text{-}e_g$ ($\Delta\varepsilon\_a_2\text{-}e$) (eV)		$\Delta\varepsilon\_a_{2u}\text{-}e_g$ ($\Delta\varepsilon\_a_1\text{-}e$) (eV)	
	gas phase	solvent	gas phase	solvent
**P**	4.82	4.84	4.95	4.93
**Pz**	4.33	4.32	6.03	6.03
**TBP**	4.13	4.15	5.16	5.12
**Pc**	3.73	3.72	6.19	6.20
**ZnSubP**	5.62	5.63	5.93	5.89
**SubP**	5.69	5.71	5.89	5.93
**SubPz**	5.26	5.25	6.93	6.98
**TBSubP**	4.92	4.92	6.03	6.06
**SubPc**	4.57	4.54	6.87	6.83

S2.3 Expanded Gouterman model to interpret B band

To include the effect of transitions other than the H-1 to L in the B band, we computed the pondered $\Delta\varepsilon$ based on the transitions involved in the B band and their respective weights.

Table S20. Calculated pondered $\Delta\varepsilon$ in eV at CAM-B3LYP/cc-pVTZ level of theory considering an implicit solvent (scrf=(pcm,solvent)). See experimental data section in the manuscript for the solvent used in each case.

system	transition	$\Delta\varepsilon$ (eV)	ponderation	pondered $\Delta\varepsilon$ (eV)
**P**	$94 \rightarrow 96$	4.93	0.52	4.89
	$95 \rightarrow 97$	4.84	0.48	
**Pz**	$88 \rightarrow 97$	6.97	0.18	6.22
	$91 \rightarrow 98$	6.54	0.11	
	$92 \rightarrow 97$	6.48	0.34	
	$93 \rightarrow 97$	6.03	0.26	
	$95 \rightarrow 96$	4.32	0.11	

**TBP**	146 → 148	5.12	0.61	4.74
	147 → 149	4.15	0.30	
	147 ← 149	4.15	0.10	
**Pc**	138 → 149	6.57	0.13	6.11
	139 → 148	6.56	0.10	
	145 → 149	6.23	0.15	
	146 → 149	6.20	0.44	
	147 → 148	3.72	0.10	
	136 → 149	7.13	0.07	
**SubP**	67 → 72	7.87	0.10	6.04
	70 → 72	5.93	0.50	
	71 → 73	5.71	0.39	
**SubPz**	67 → 72	7.60	0.22	6.81
	70 → 72	6.98	0.61	
	71 → 73	5.25	0.18	
**TBSubP**	109 → 111	6.06	0.72	5.74
	110 → 112	4.92	0.28	
**SubPc**	104 → 111	7.41	0.13	6.65
	107 → 111	6.83	0.55	
	108 → 111	6.77	0.11	
	109 → 112	6.77	0.11	
	110 → 112	4.54	0.11	

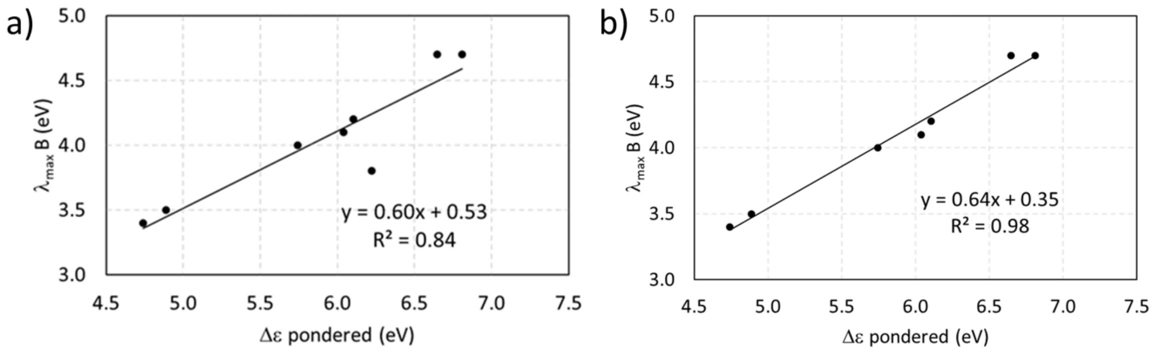

Figure S4. Relationship between B band λ_{max} and pondered Δε in eV including a) all systems and b) including all systems but **Pz**.

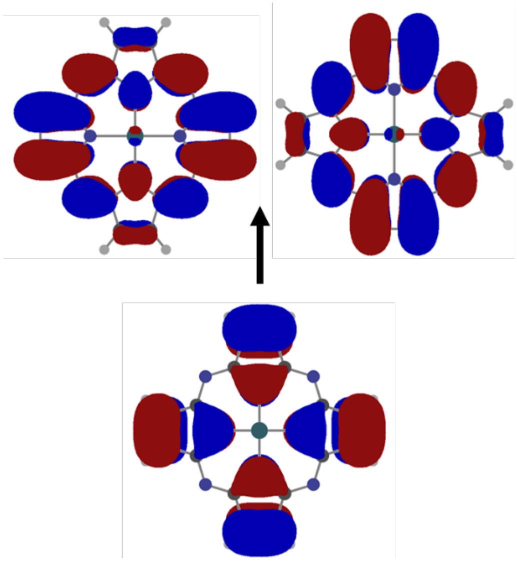

Figure S5. Representation of the **Pz** orbitals involved in the $b_{2u} \rightarrow e_g$ (92 → 97,98) transition. The isocontour is 0.02.

S3. Singlet-Triplet Energy Gap

Table S21. $\Delta E_{T_x \rightarrow S_1}$ values computed at TD and TDA with CAM-B3LYP/cc-pVTZ in gas phase level of theory. In all cases, the reference S_1 energies taken correspond to the TD formalism and the degenerate T_1/T_2 (labeled T_1) and T_3/T_4 (labeled T_2) energies have been calculated at both, TD and TDA levels.

	TD		TDA	
	$\Delta E_{T_1 \rightarrow S_1}$	$\Delta E_{T_2 \rightarrow S_1}$	$\Delta E_{T_1 \rightarrow S_1}$	$\Delta E_{T_2 \rightarrow S_1}$
**P**	-1.09	-0.23	-0.47	-0.21
**Pz**	-1.48	0.41	-0.92	0.62
**TBP**	-1.05	0.10	-0.58	0.23
**Pc**	-1.55	1.04	-0.91	1.28
**SubP**	-1.12	-0.44	-0.66	-0.40
**SubPz**	-1.41	0.14	-1.03	0.24
**TBSubP**	-1.04	0.04	-0.71	0.18
**SubPc**	-1.34	0.63	-0.96	0.96

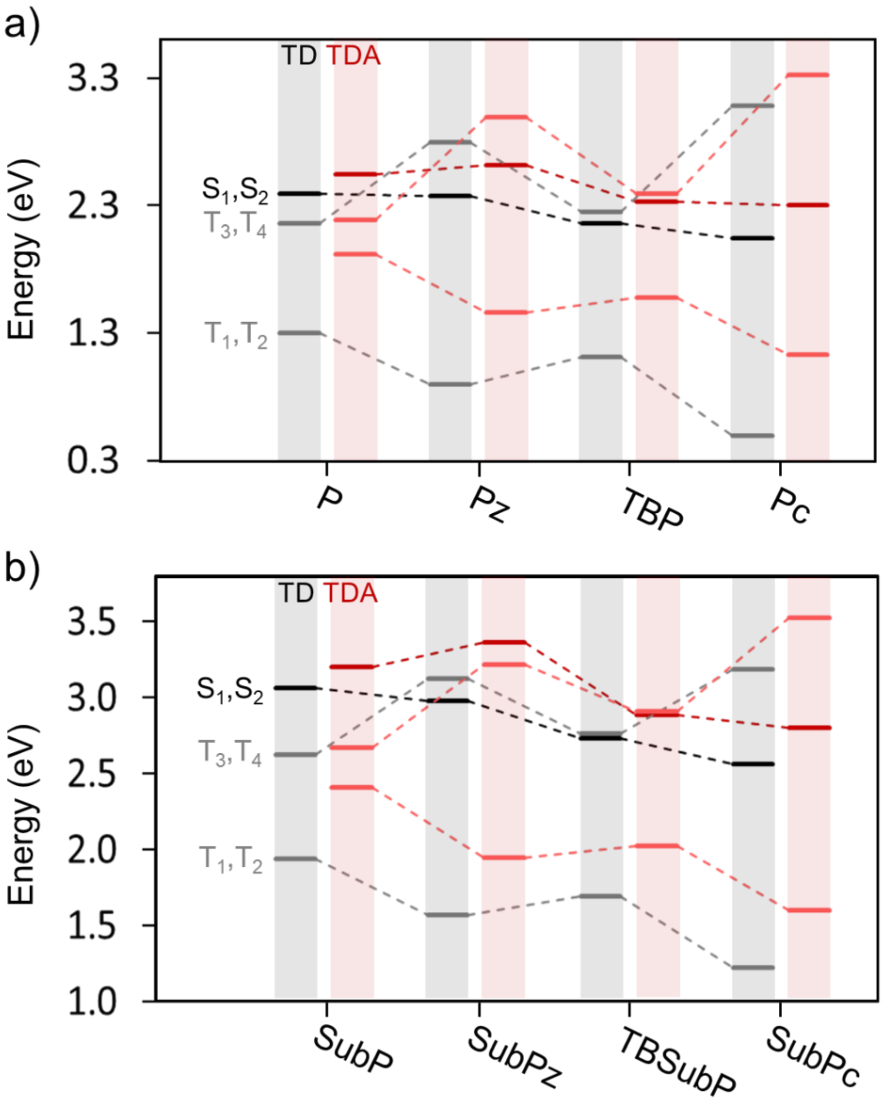

Figure S6. Excitation energies for the low lying singlet (dark) and triplet (light) states at TD (black) and TDA (red) with CAM-B3LYP/cc-pVTZ in gas phase level of theory, of a) D_{4h} and b) C_{3v} molecules. In both cases, the singlets and triplets shown correspond to excited states of E_u (or E in the case of C_{3v} systems) symmetry.

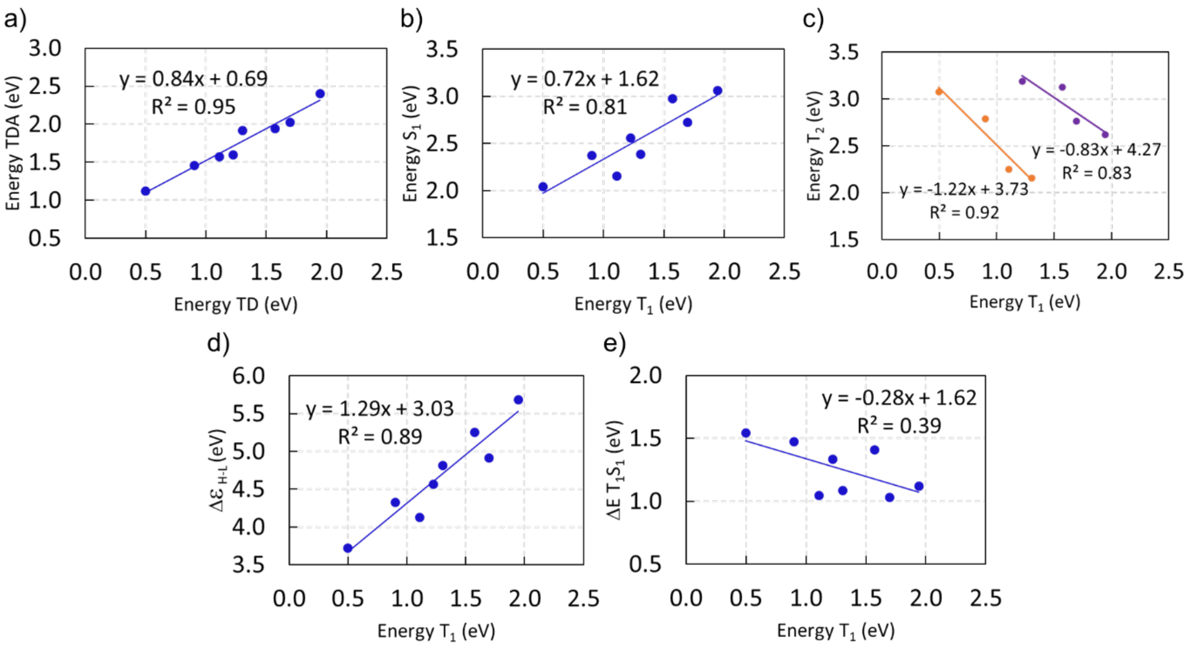

Figure S7. Comparative energy correlations: a) T_1 energies via TDA-TDDFT and TDDFT methodologies, b) Relationship between S_1 (Q band) and T_1 states at TDDFT level of theory, c) triplet state energies divided into D_{4h} (orange) and C_{3v} (purple) groups for initial roots (T_1) versus subsequent roots (T_2) at TDDFT level of theory, d) T_1 energy against the $\Delta\varepsilon_{HOMO-LUMO}$ or $\Delta\varepsilon$_a$_{1u}$-e$_g$ ($\Delta\varepsilon$_a$_2$-e for C_{3v} systems), e) T_1 energy against the $\Delta E_{T_1 \rightarrow S_1}$.

S4. Electron Delocalization and Aromaticity Measures

S4.1. GIMIC

The magnetically induced current density calculations were computed with the GIMIC (gauge-including magnetically induced currents) program available at GitHub (https://gimic.readthedocs.io). The input data required for running GIMIC are the one-electron density matrix, the magnetically perturbed one-electron matrices and basis set information and were obtained from an NMR shielding calculation (NMR=GIAO, Int=NoBasisTransform, and IOp(10/33=2) keywords) performed with Gaussian 09 rev. D.01. For the calculation of the current density plots and the current strengths, we had to set the following parameters: integration plane, orientation of the magnetic field, dimensions of the cube (volume in which the calculation is done, defined by the origin and length) and the grid spacing. One has to note that GIMIC uses Bohr length unit, thus all the parameters involving distances will be in this unit. The magnetic field was oriented perpendicular to the molecular plane, *xy* (ivect=[1,0,0] and jvect=[0,1,0]), pointing towards the *z* direction. In the particular case of the planar structures, the choice was clear. However, for the non-planar subporphyrins and subpthalocyanines, we used different orientations of the magnetic field (Figure S8a) to estimate the induced currents. The

grid origin, spacing of the points, and cube length have been defined depending on the size of the molecule to make sure that all faces of the cube are 8 bohr apart from the carbon skeleton of the molecule (Figure S8b). Finally, the current densities were visualized using ParaView 5.9.0[22] and the line integral convolution technique (LIC).

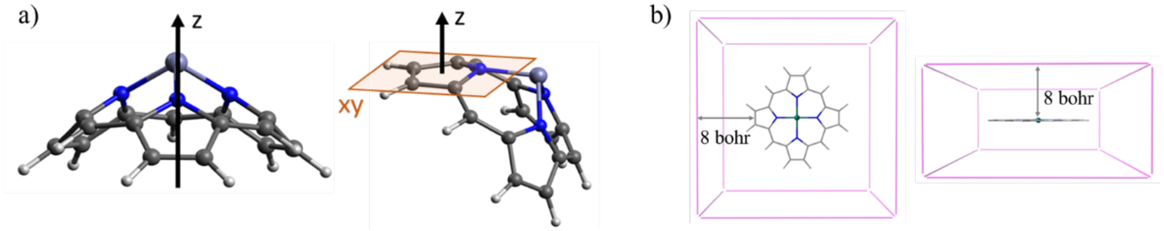

Figure S8. Representation of the a) orientations selected for the non-planar molecules b) cube dimensions.

For the calculation of bond currents, integration planes perpendicular to the bonds were defined. The integration was done with the Gauss quadrature and specifying the following keywords: gauss_order=9, grid_points=[100, 100, 0], height=[-5.0, 5.0], width=[*in*, *out*]. The height of the planes was set to 5 bohr above and below the bond and the *in* and *out* values of the width were chosen depending on the geometrical center of the rings. The out distance has been set to 8 Bohrs in all the cases except for situations like bond 2-3 or 4-5 (Figure S9), where the plane starts and ends at the geometrical center of another ring.

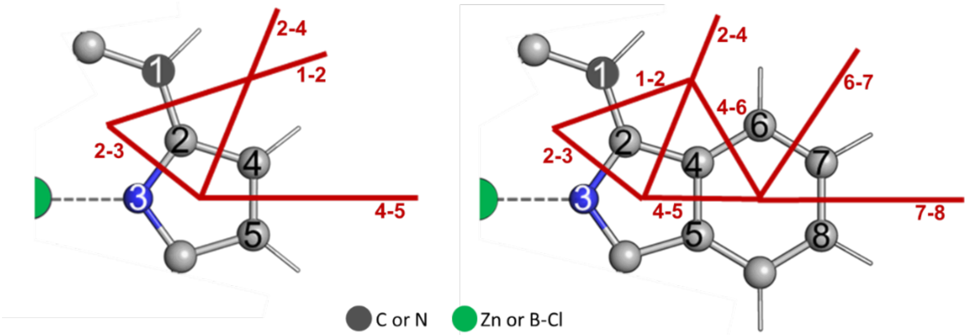

Figure S9. Representation of the integration planes used for the calculation of bond currents.

From the GIMIC plots, we obtained both qualitative (current densities) and semiquantitative (current strengths) results. According to the convention, in the current density plots the current flowing clockwise is diatropic (and its integrated current density is positive), while a counterclockwise current is paratropic (the integrated current density is negative). In the current density plots, the intensity of the current decreases going from white (0.4 $nA\cdot T^{-1}\cdot Å^{-2}$) or light yellow to dark red and black ($4\cdot 10^{-5}$ $nA\cdot T^{-1}\cdot Å^{-2}$), and the black arrows indicate the direction of the current flow.

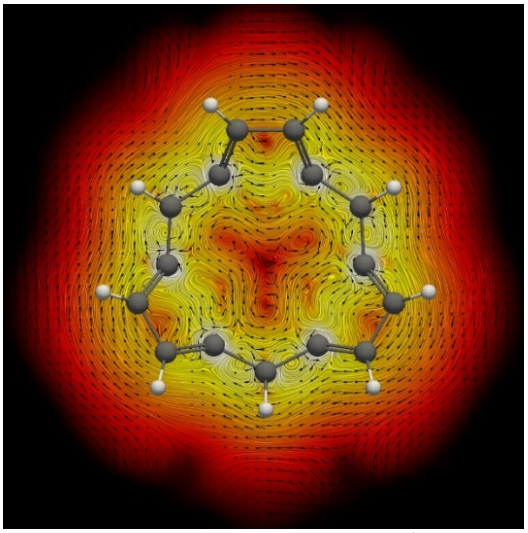

SubP(Zn): (meso C-C-C *xy* plane)

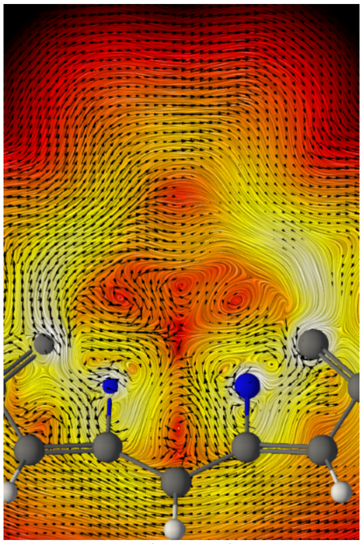

(pyrrole *xy* plane)

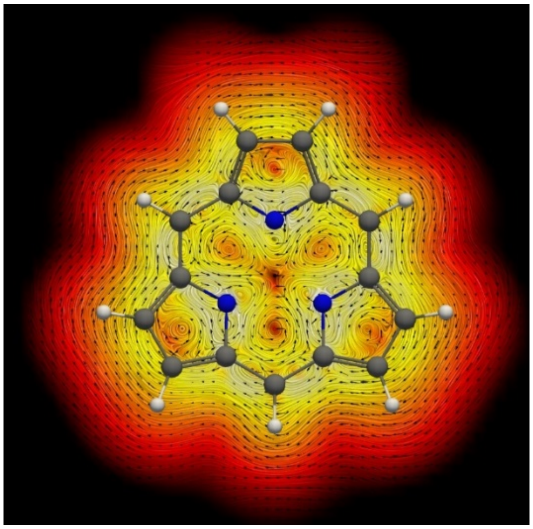

SubP: (meso C-C-C *xy* plane)

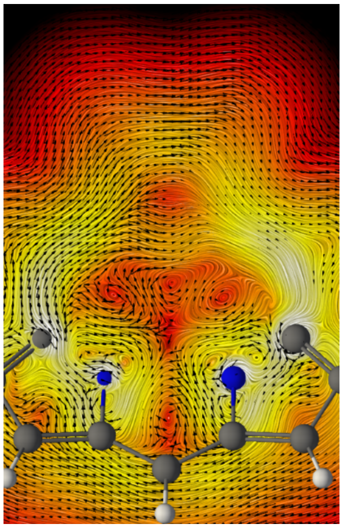

(pyrrole *xy* plane)

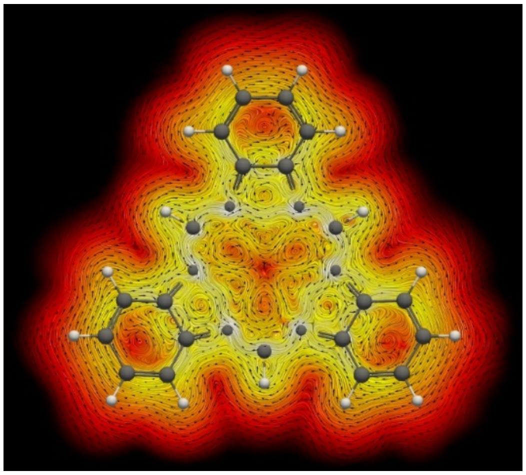

TBSubP: (C-C-C *xy* plane)

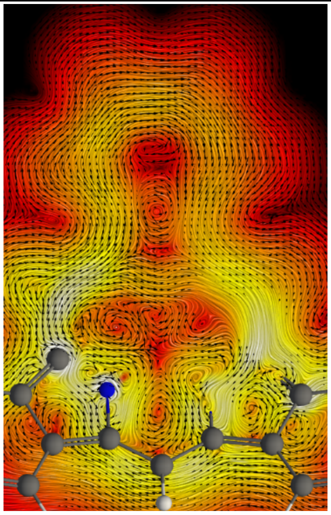

(isoindole *xy* plane)

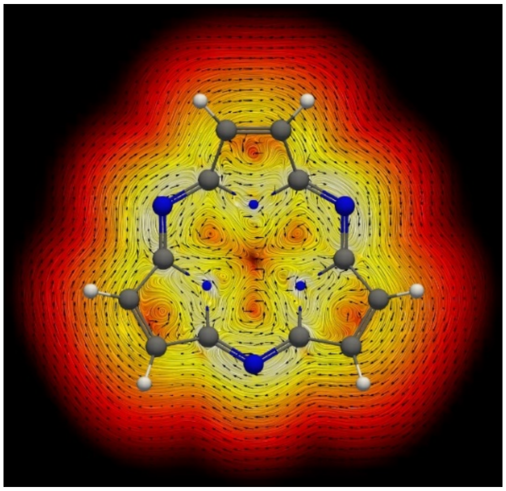

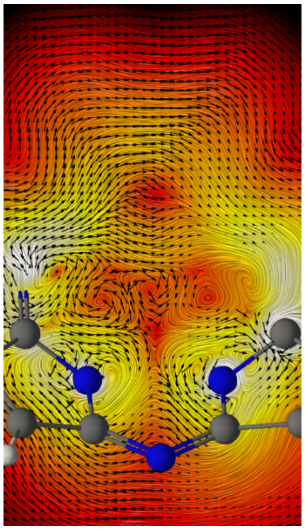

SubPz: (meso N-N-N *xy* plane) (pyrrole *xy* plane)

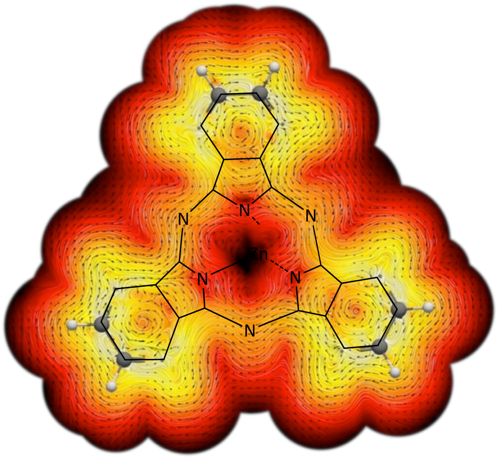

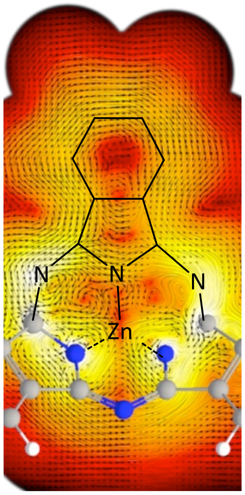

SubPc: (meso N-N-N *xy* plane) (isoindole *xy* plane)

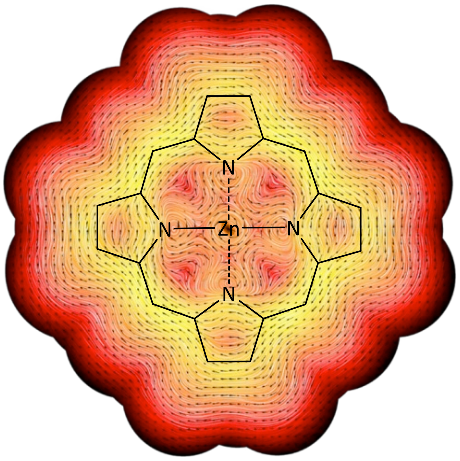

P

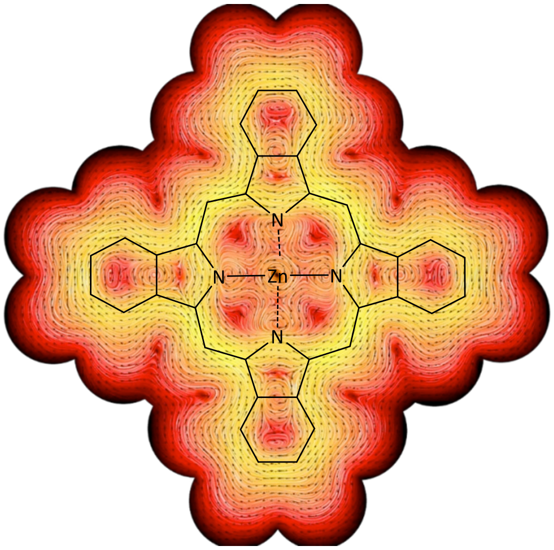

TBP

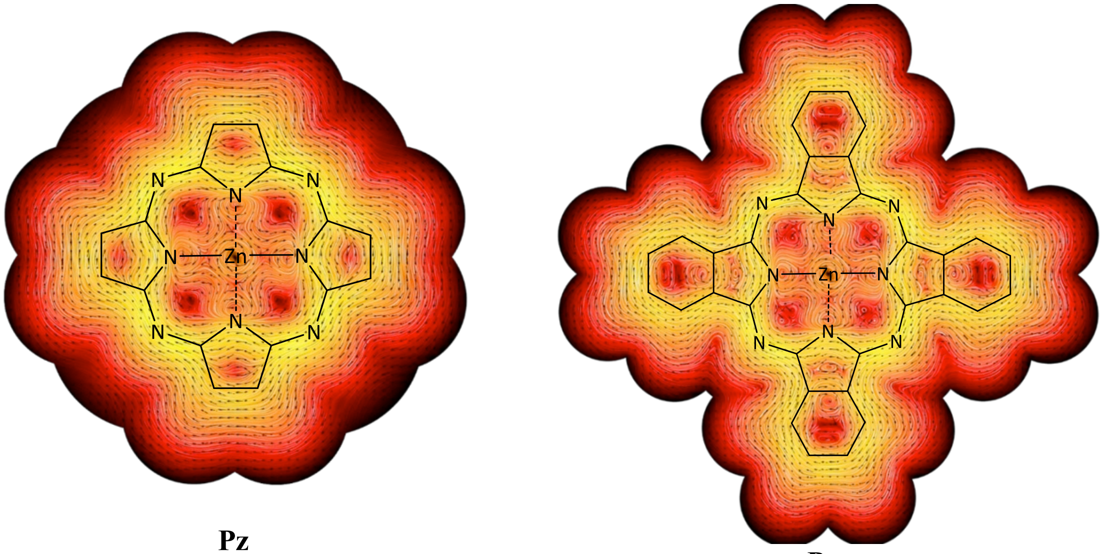

Pz **Pc**

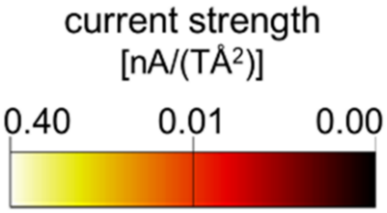

Figure S10. Current density in the S_0 state visualized using the LIC representation. For systems **SubP-Zn** (x=1.5), **SubP** (x=1.0), **TBSubP** (x=1.5), **SubPz** (x=1.0) and **SubPc** (x=2.0) the visualized plane on left side is located at x Å below the Zn/B atom and the plane on the right is 1 Å below the pyrrole/isoindole ring. For planar systems **P**, **TBP**, **TPz** and **Pc** the plane is 1 Å above the molecular plane. The color scale corresponds to the strength of the modulus of the current-density susceptibility in the range of 0.00 (black) to 0.40 (white) nA/T/Å^2.

S4.2. HOMA, FLU, AV1245, AV$_{min}$, and EDDB

In the manuscript, we also computed additional measures of aromaticity such as the harmonic oscillator measure of aromaticity (HOMA)[23] and the fluctuation index (FLU),[24,25] , which are not included in the manuscript for the sake of simplicity. The latter measures are both based on comparing the system of interest with a reference aromatic molecule. In the case of C-C bonds, the reference is benzene, while in the case of C-N bonds, the reference is pyridine. In the case of HOMA, values close to 1 are indicative of aromaticity while smaller values are indicative of non- or antiaromaticity. In the case of FLU, values close to 0 correspond to aromatic rings. In the case of AV1245,[26] AV$_{min}$,[27] and multicenter indices[28,29] no references are needed and the larger the value, the more aromatic the compound is. The calculation of the electron density of delocalized bonds (EDDB)[30-32] is also done

without the use of internal references. There are different variants of the EDDB function, here we are going to discuss mainly the $EDDB_P$, which estimates the electron delocalization along the selected pathway of adjacent bonds. For this index, the larger the normalized value according to the number of atoms, the more aromatic the compound/pathway is. On Table S21, we compile the values of the different indices previously mentioned for some aromatic, non-aromatic and antiaromatic reference systems.

Table S22. Aromaticity results for some annulenes, C_6H_{12} and pyrrole, computed at CAM-B3LYP/cc-pVTZ level of theory. CBD stands for cyclobutadiene.

**System**	FLU	HOMA	MCI	$MCI^{1/N}$	AV1245	AV_{min}
benzene	0.0000	1.002	0.071	0.643	10.50	10.50
CBD	0.1009	-3.679	0.010	0.317	-	-
C_6H_{12}	0.0895	-3.888	0.000	0.261	-0.01	-0.01
pyrrole	0.0053	0.871	0.039	0.524	-	-
$C_{16}H_{16}$ (S4)	0.0399	0.157	-	-	0.75	0.63
$C_{18}H_{18}$ (TS)	0.0005	0.985	-	-	4.31	4.23

**system**	$EDDB_G$	$EDDB_G$/atom	$EDDB_H$	$EDDB_H$/atom	$EDDB_P$	$EDDB_P$/atom	up. $Limit_P$	up. $Limit_P$/atom
benzene	6.160	0.513	5.618	0.936	5.492	0.915	5.492	0.915
CBD	0.381	0.048	0.197	0.049	0.099	0.025	0.099	0.025
C_6H_{12}	1.261	0.070	0.377	0.063	0.323	0.054	0.323	0.054
pyrrole	4.077	0.408	3.759	0.752	2.950	0.590	2.901	0.580
$C_{16}H_{16}$ (S4)	5.754	0.180	4.185	0.262	1.584	0.099	1.568	0.098
$C_{18}H_{18}$ (TS)	19.996	0.555	18.546	1.030	14.882	0.827	14.776	0.821

We want to determine if there is a preferred circuit (in terms of electron delocalization) among all different circuits present in the molecule. Thus, we need to consider all the possible pathways and compute the aromaticity of each of them. The pathways in **SubP**, **SubPz**, **P** and **Pz**, are labelled according to the nomenclature used in the 2018 study of porphyrinoids by Casademont-Reig *et al.*,[20] starting from the pyrrolic ring on the top (Figure S11). In the case of the remaining systems (phthalocyanine analogues) the "o i" (where o and i stands for *outer* and *inner*, respectively) nomenclature is maintained in the circuits passing through the C-C or C-N-C bonds of the pyrrolic units, and we used "b" (were "b" stands for *benzo*) to indicate the pass through the outer part of the 6-MR rings.

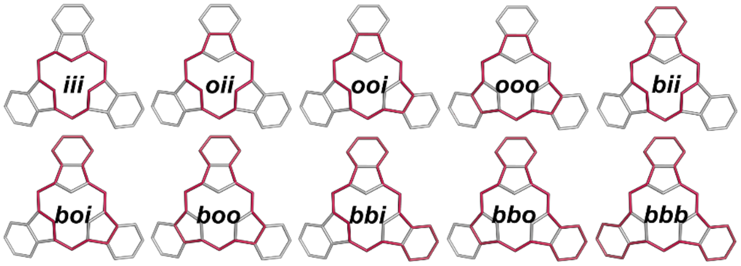

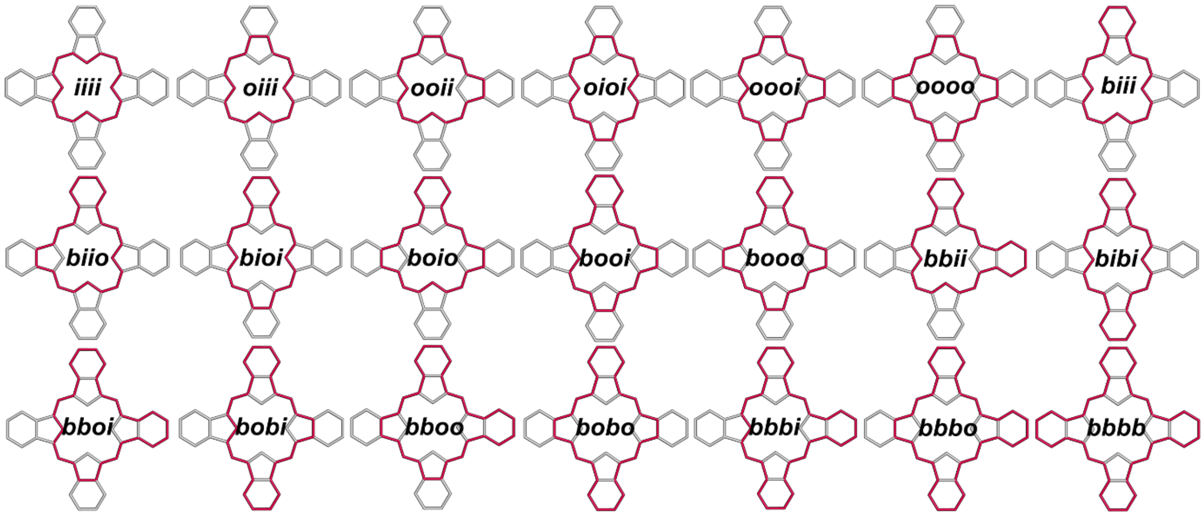

Figure S11. Representation of the different pathways considered.

The aromaticity results obtained with the previously mentioned indices for the systems in the S_0 state are summarized in the following tables. The values in bold and green colour correspond to the most aromatic pathway, other values highlighted in green correspond to similarly aromatic pathways.

Table S23. Aromaticity indices for different pathways in **P** system, computed at CAM-B3LYP/cc-pVTZ level of theory.

Pathway[a]	*N*	FLU	HOMA	AV1245	AV_{min}	$EDDB_p$[b]	upper $limit_p$[b]
iiii	16	**0.003**	**0.965**	0.99	0.42	0.652	0.560
oiii (4)	17	0.008	0.878	1.20	0.42	0.565	0.260
ooii (4)	18	0.013	0.801	1.39	0.42	0.487	0.258
oioi (2)	18	0.013	0.801	1.39	0.42	0.487	0.259
oooi (4)	19	0.017	0.731	1.56	0.42	0.418	0.255
oooo	20	0.021	0.669	1.72	0.51	0.355	0.255

[a]The value in parenthesis indicates the number of equivalent pathways. [b]The values are normalized with respect the number of atoms forming the circuit (*N*).

Table S24. Aromaticity indices for different pathways in **Pz** system, computed at CAM-B3LYP/cc-pVTZ level of theory.

Pathway[a]	*N*	FLU	HOMA	AV1245	AV_{min}	$EDDB_p$[b]	upper $limit_p$[b]
iiii	16	**0.003**	**0.973**	**1.27**	**0.90**	**0.732**	**0.554**
oiii (4)	17	0.012	0.829	1.24	0.50	0.606	0.157
ooii (4)	18	0.020	0.701	1.21	0.50	0.494	0.153
oioi (2)	18	0.020	0.701	1.21	0.50	0.493	0.155
oooi (4)	19	0.027	0.586	1.19	0.50	0.393	0.150
oooo	20	0.034	0.483	1.16	0.50	0.303	0.148

[a]The value in parenthesis indicates the number of equivalent pathways. [b]The values are normalized with respect the number of atoms forming the circuit (*N*).

Table S25. Aromaticity indices for different pathways in **TBP** system, computed at CAM-B3LYP/cc-pVTZ level of theory.

Pathway[a]	N	FLU	HOMA	AV1245	AV_{min}	$EDDB_p$[b]	upper $limit_p$[b]
iiii	16	**0.002**	**0.956**	1.04	0.57	**0.680**	**0.587**
oiii (4)	17	0.009	0.855	0.99	0.57	0.566	0.197
ooii (4)	18	0.015	0.766	0.97	0.28	0.465	0.195
oioi (2)	18	0.015	0.766	0.95	0.57	0.465	0.196
oooi (4)	19	0.021	0.686	0.95	0.28	0.375	0.193
oooo	20	0.026	0.614	0.94	0.28	0.294	0.192
biii (4)	21	0.008	0.879	1.67	0.57	0.609	0.343
biio (8)	22	0.013	0.805	1.62	0.28	0.524	0.196
bioi (4)	22	0.013	0.805	1.61	0.57	0.524	0.197
boio (4)	23	0.017	0.737	1.57	0.28	0.447	0.195
booi (8)	23	0.017	0.737	1.57	0.28	0.447	0.195
booo (4)	24	0.022	0.675	1.54	0.28	0.377	0.193
bbii (4)	26	0.011	0.832	2.08	0.28	0.565	0.318
bibi (2)	26	0.011	0.832	2.06	0.57	0.565	0.341
bboi (8)	27	0.015	0.773	2.02	0.28	0.498	0.195
bobi (4)	27	0.015	0.773	2.02	0.28	0.498	0.194
bboo (4)	28	0.019	0.719	1.98	0.28	0.435	0.193
bobo (2)	28	0.019	0.719	1.98	0.28	0.435	0.193
bbbi (4)	31	0.013	0.800	2.35	0.28	0.535	0.315
bbbo (4)	32	0.017	0.751	2.30	0.28	0.480	0.194
bbbb	36	0.015	0.776	**2.55**	0.28	0.514	0.313

[a]The value in parenthesis indicates the number of equivalent pathways. [b]The values are normalized with respect the number of atoms forming the circuit (N).

Table S26. Aromaticity indices for different pathways in **Pc** system, computed at CAM-B3LYP/cc-pVTZ level of theory.

Pathway[a]	N	FLU	HOMA	AV1245	AV_{min}	$EDDB_p$[b]	upper $limit_p$[b]
iiii	16	**0.003**	**0.959**	1.06	**0.60**	**0.751**	**0.566**
oiii (4)	17	0.013	0.829	0.90	0.40	0.614	0.153
ooii (4)	18	0.022	0.713	0.76	0.40	0.493	0.150
oioi (2)	18	0.022	0.713	0.76	0.40	0.493	0.152
oooi (4)	19	0.030	0.609	0.64	0.40	0.385	0.148
oooo	20	0.037	0.516	0.53	0.40	0.288	0.147
biii (4)	21	0.011	0.860	1.65	0.44	0.652	0.310
biio (8)	22	0.018	0.763	1.51	0.40	0.551	0.151
bioi (4)	22	0.018	0.763	1.50	0.40	0.550	0.152
boio (4)	23	0.025	0.676	1.37	0.40	0.458	0.150
booi (8)	23	0.025	0.676	1.37	0.40	0.459	0.149
booo (4)	24	0.031	0.595	1.25	0.40	0.374	0.148
bbii (4)	26	0.015	0.799	2.02	0.40	0.591	0.281
bibi (2)	26	0.015	0.799	2.02	0.44	0.590	0.307
bboi (8)	27	0.021	0.722	1.89	0.40	0.510	0.150
bobi (4)	27	0.021	0.722	1.89	0.40	0.510	0.150
bboo (4)	28	0.026	0.652	1.77	0.40	0.436	0.148
bobo (2)	28	0.026	0.652	1.77	0.40	0.436	0.148
bbbi (4)	31	0.018	0.757	2.27	0.40	0.549	0.277
bbbo (4)	32	0.023	0.694	2.15	0.40	0.483	0.149

bbbb	36	0.020	0.727	**2.45**	0.40	0.519	0.274

[a]The value in parenthesis indicates the number of equivalent pathways. [b]The values are normalized with respect the number of atoms forming the circuit (*N*).

Table S27. Aromaticity indices for different pathways in **Zn-SubP** system, computed at CAM-B3LYP/cc-pVTZ level of theory.

Pathway[a]	*N*	FLU	HOMA	AV1245	AV_{min}	$EDDB_p$[b]	upper $limit_p$[b]
iii	12	**0.005**	**0.900**	0.70	0.24	**0.605**	**0.523**
oii (3)	13	0.010	0.796	1.28	0.24	0.533	0.335
ooi (3)	14	0.014	0.707	1.78	0.24	0.472	0.334
ooo	15	0.018	0.629	**2.22**	**0.94**	0.419	0.332

[a]The value in parenthesis indicates the number of equivalent pathways. [b]The values are normalized with respect the number of atoms forming the circuit (*N*).

Table S28. Aromaticity indices for different pathways in **SubP** system, computed at CAM-B3LYP/cc-pVTZ level of theory.

Pathway[a]	*N*	FLU	HOMA	AV1245	AV_{min}	$EDDB_p$[b]	upper $limit_p$[b]
iii	12	**0.008**	**0.959**	0.80	0.24	**0.566**	**0.499**
oii (3)	13	0.011	0.874	1.37	0.24	0.516	0.354
ooi (3)	14	0.014	0.802	1.88	0.24	0.472	0.353
ooo	15	0.017	0.739	**2.33**	**0.94**	0.435	0.352

[a]The value in parenthesis indicates the number of equivalent pathways. [b]The values are normalized with respect the number of atoms forming the circuit (*N*).

Table S29. Aromaticity indices for different pathways in **SubPz** system, computed at CAM-B3LYP/cc-pVTZ level of theory.

Pathway[a]	*N*	FLU	HOMA	AV1245	AV_{min}	$EDDB_p$[b]	upper $limit_p$[b]
iii	12	**0.011**	**0.976**	0.40	0.05	**0.632**	**0.494**
oii (3)	13	0.017	0.847	0.94	0.05	0.537	0.250
ooi (3)	14	0.022	0.737	1.40	0.05	0.454	0.247
ooo	15	0.026	0.642	**1.80**	**1.32**	0.382	0.245

[a]The value in parenthesis indicates the number of equivalent pathways. [b]The values are normalized with respect the number of atoms forming the circuit (*N*).

Table S30. Aromaticity indices for different pathways in **TBSubP** system, computed at CAM-B3LYP/cc-pVTZ level of theory.

Pathway[a]	*N*	FLU	HOMA	AV1245	AV_{min}	$EDDB_p$[b]	upper $limit_p$[b]
iii	12	**0.007**	**0.967**	0.61	0.02	**0.597**	**0.530**
oii (3)	13	0.014	0.831	0.84	0.02	0.501	0.250
ooi (3)	14	0.019	0.715	1.04	0.02	0.420	0.248
ooo	15	0.024	0.615	1.22	0.50	0.349	0.247
bii(3)	17	0.011	0.876	1.71	0.02	0.570	0.383
boi(6)	18	0.015	0.784	1.82	0.02	0.502	0.249
boo(3)	19	0.019	0.701	1.92	0.50	0.442	0.247
bbi(3)	22	0.012	0.827	2.31	0.02	0.555	0.366
bbo(3)	23	0.016	0.757	2.38	0.50	0.503	0.249
bbb	27	0.014	0.796	**2.70**	0.50	0.545	0.366

[a]The value in parenthesis indicates the number of equivalent pathways. [b]The values are normalized with respect the number of atoms forming the circuit (*N*).

Table S31. Aromaticity indices for different pathways in **SubPc** system, computed at CAM-B3LYP/cc-pVTZ level of theory.

Pathway[a]	N	FLU	HOMA	AV1245	AV_{min}	$EDDB_p$[b]	upper $limit_p$[b]
iii	12	**0.011**	**0.973**	0.59	0.36	**0.661**	**0.517**
oii (3)	13	0.019	0.811	0.69	0.36	0.538	0.218
ooi (3)	14	0.027	0.672	0.77	0.36	0.434	0.215
ooo	15	0.033	0.552	0.83	0.65	0.343	0.214
bii(3)	17	0.015	0.863	1.66	0.36	0.599	0.344
boi(6)	18	0.021	0.752	1.67	0.36	0.514	0.216
boo(3)	19	0.026	0.653	1.67	0.65	0.438	0.214
bbi(3)	22	0.017	0.803	2.24	0.36	0.565	0.323
bbo(3)	23	0.021	0.719	2.22	0.65	0.500	0.216
bbb	27	0.018	0.765	**2.60**	0.65	0.544	0.321

[a]The value in parenthesis indicates the number of equivalent pathways. [b]The values are normalized with respect the number of atoms forming the circuit (N).

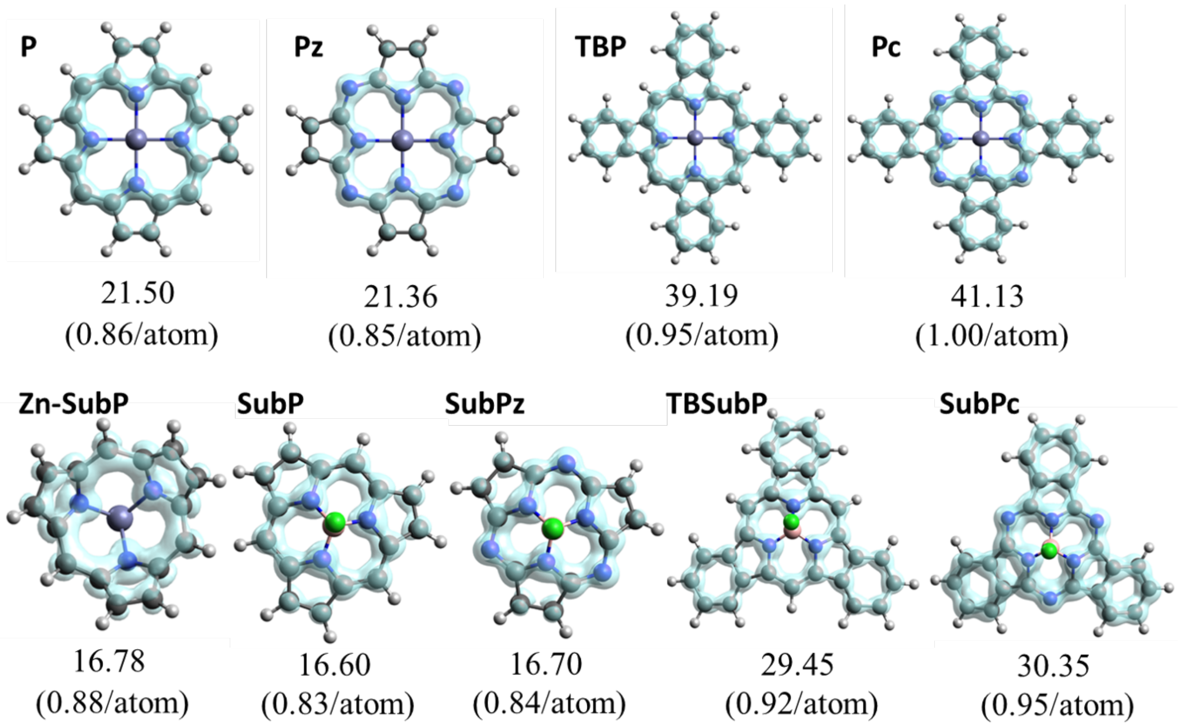

Figure S12. Representation of the $EDDB_H$ surface (isovalue 0.03e, visualized using Avogadro version 1.2.0)[33] and the corresponding value of delocalized electrons in the S_0 state of **P**, **Pz**, **TBP**, **Pc**, **Zn-SubP**, **SubP**, **SubPz**, **TBSubP**, and **SubPc** systems at CAM-B3LYP/cc-pVTZ level of theory.

We also evaluated the local aromaticity of the individual 5 and 6-member rings (MR) present in the molecule.

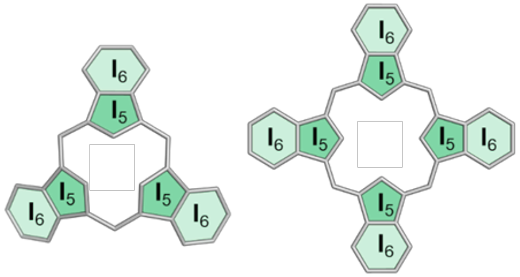

Figure S13. Types of 5- and 6-MR present in the studied systems.

Table S32. Aromaticity indices for 5-MR (**I_5**) in studied systems, computed at CAM-B3LYP/cc-pVTZ level of theory in S_0 state.

system	FLU	HOMA	I_{ring}	$I_{ring}^{1/N\ a}$	MCI	$MCI^{1/N\ a}$	$EDDB_p$	$EDDB_p^b$	upper $limit_p$	upper $limit_p^b$
**SubP(Zn)**	0.018	0.648	0.021	0.462	0.022	0.467	2.098	0.420	1.564	0.313
**SubP**	0.019	0.731	0.020	0.457	0.020	0.459	2.077	0.415	1.538	0.308
**TBSubP**	0.027	0.600	0.012	0.413	0.011	0.407	1.789	0.358	1.341	0.268
**SubPz**	0.034	0.623	0.018	0.450	0.018	0.448	1.732	0.346	1.240	0.248
**SubPc**	0.040	0.532	0.012	0.410	0.011	0.405	1.561	0.312	1.110	0.222
**P**	0.019	0.642	0.023	0.470	0.023	0.471	1.903	0.381	1.344	0.269
**TBP**	0.025	0.592	0.014	0.427	0.013	0.420	1.575	0.315	1.008	0.202
**Pz**	0.036	0.468	0.020	0.456	0.018	0.447	1.431	0.286	0.777	0.155
**Pc**	0.039	0.499	0.013	0.418	0.011	0.409	1.304	0.261	0.750	0.150
**pyrrole**	0.005	0.871	0.028	0.490	0.039	0.524	2.950	0.590	2.901	0.580

[a] N is the number of atoms in the ring, N=5. [b] $EDDB_P$ results per atom.

Table S33. Aromaticity indices for 6-MR (**I_6**) in studied systems, computed at CAM-B3LYP/cc-pVTZ level of theory in S_0 state.

system	FLU	HOMA	I_{ring}	$I_{ring}^{1/N\ a}$	MCI	$MCI^{1/N\ a}$	AV1245	AV_{min}	$EDDB_p$	$EDDB_p^b$	upper limit	upper limit[b]
**TBSubP**	0.004	0.948	0.033	0.567	0.048	0.603	7.38	5.81	4.233	0.705	3.580	0.597
**SubPc**	0.004	0.955	0.034	0.570	0.050	0.606	7.50	5.99	4.315	0.719	3.745	0.624
**TBP**	0.004	0.981	0.034	0.570	0.050	0.607	7.58	6.13	4.313	0.719	3.710	0.618
**Pc**	0.003	0.989	0.036	0.574	0.052	0.611	7.79	6.46	4.442	0.740	3.928	0.655
**benzene**	0.000	1.002	0.047	0.601	0.071	0.643	10.50	10.50	5.492	0.915	5.491	0.915

[a] N is the number of atoms in the ring, N=6. [b] $EDDB_P$ results per atom.

S4.3. N-Center Delocalization Indices (δ_{2c} and δ_{4c})

The delocalization indices (DI or δ_{2c}) at the CAM-B3LYP/cc-pVTZ level of theory for the studied systems are presented in the following figure. Reference DIs for isolated benzene, pyrrole, cyclohexane and ethylene at the same level of theory are: benzene: $DI_{c\text{-}c}$=1.394, pyrrole: $DI_{C\text{-}N}$= 1.113, $DI_{C2\text{-}C3}$= 1.475 and $DI_{C3\text{-}C4}$=1.282, cyclohexane: $DI_{c\text{-}c}$=0.981 and ethylene: $DI_{c\text{-}c}$=1.907.

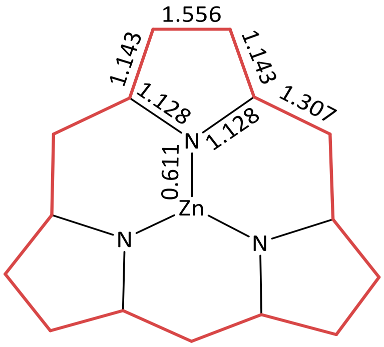

Subporphyrin-Zn (SubP(Zn))

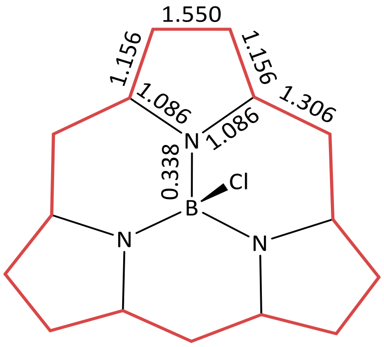

Subporphyrin (SubP)

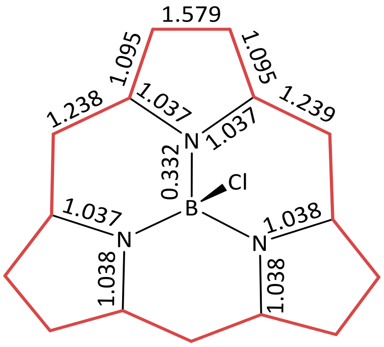

Subporphyrazine (SubPz)

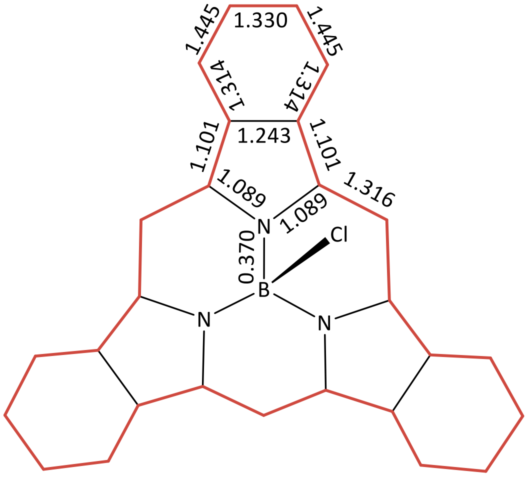

Tribenzosubporphyrin (TBSubP)

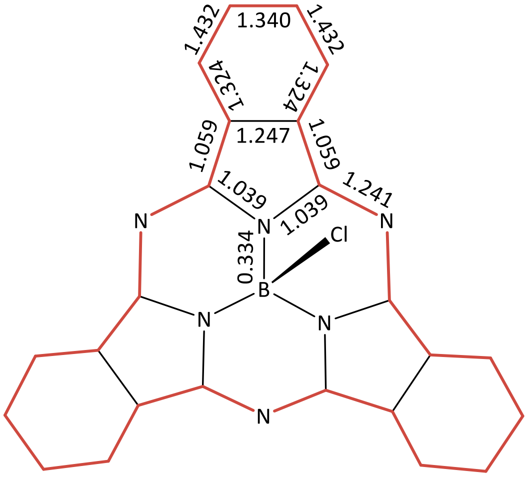

Subphthalocyanine (SubPc)

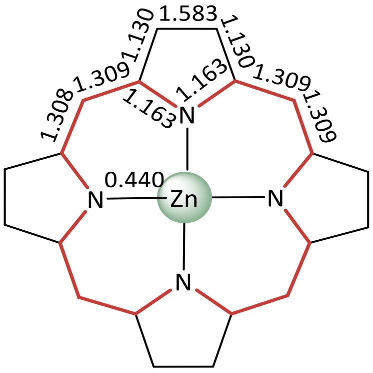

Porphyrin (P)

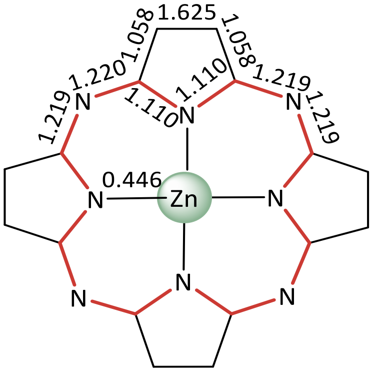

Porphyrazine (Pz)

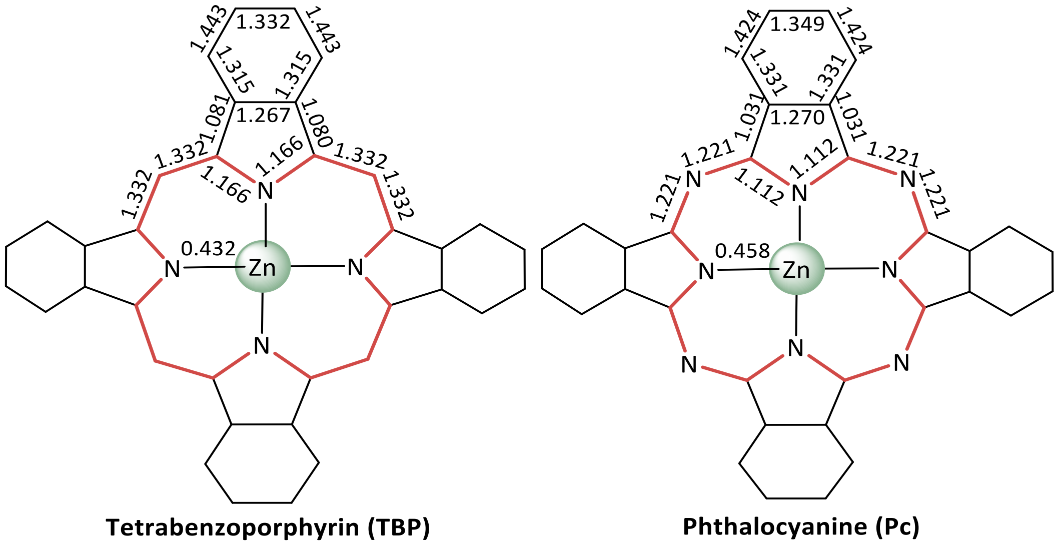

Figure S14. Delocalization indices in studied porphyrinoids and phthalocyanine analogous in the S_0 state. Conjugation pathway resulting from following the highest DI path is highlighted in bold red colored bonds.

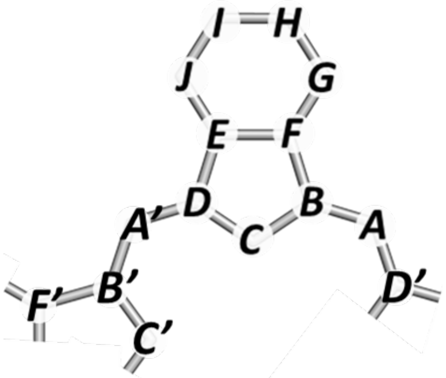

Figure S15. Labels for the different atomic positions used to compute *n* center-ESI.

Table S34. 4-c ESI, considering atoms in 1245 positions, for different systems studied. The pathway column indicates to which path the 4c-index participates. For simplification iii(i)=*i*, ooo(o)=*o*, and bbb(b)=*b*. The values used to compute AV1245 have been multiplied by a factor of 1000/3.

1245-δ(4c) pyrrolic					
δ(X,X,X,X)	**P**	**Pz**	**SubP**	**SubPz**	Pathway
**A,B,D,E**	0.0069	0.0015	0.0092	0.0040	*o,b*
**A,B,D,A'**	0.0013	0.0061	0.0049	0.0019	*i*
**B,C,A',B'**	0.0034	0.0027	0.0007	0.0001	*i*
**C,D,B',C'**	0.0037	0.0037	0.0029	0.0027	*i*
**E,D,B'F'**	0.0015	0.0021	0.0028	0.0040	*o,b*
**F,E,A',B'**	0.0053	0.0062	0.0069	0.0076	*o*

1245-δ(4c) isoindolic					
δ(X,X,X,X)	**TBP**	**Pc**	**TBSubP**	**SubPc**	Pathway
**A,B,D,E**	0.0044	0.0012	0.0057	0.0027	*o,b*
**A,B,D,A'**	0.0017	0.0054	0.0038	0.0019	*i,o,b*
**B,C,A',B'**	0.0032	0.0018	0.0001	0.0011	*i*
**C,D,B',C'**	0.0044	0.0038	0.0035	0.0030	*i*
**D,A',C'D'**	0.0032	0.0018	0.0001	0.0011	*i*
**E,D,B'F'**	0.0009	0.0012	0.0015	0.0020	*o,b*
**F,E,A',B'**	0.0023	0.0022	0.0027	0.0025	*o*
**E,D,B',C'**	0.0021	0.0023	0.0023	0.0026	*o,b*
**A,B,G,H**	0.0027	0.0013	0.0034	0.0023	*b*
**B,F,H,I**	0.0045	0.0035	0.0052	0.0044	*b*
**F,G,I,J**	0.0249	0.0254	0.0245	0.0248	*b*
**J,E,A',B'**	0.0019	0.0023	0.0025	0.0027	*b*

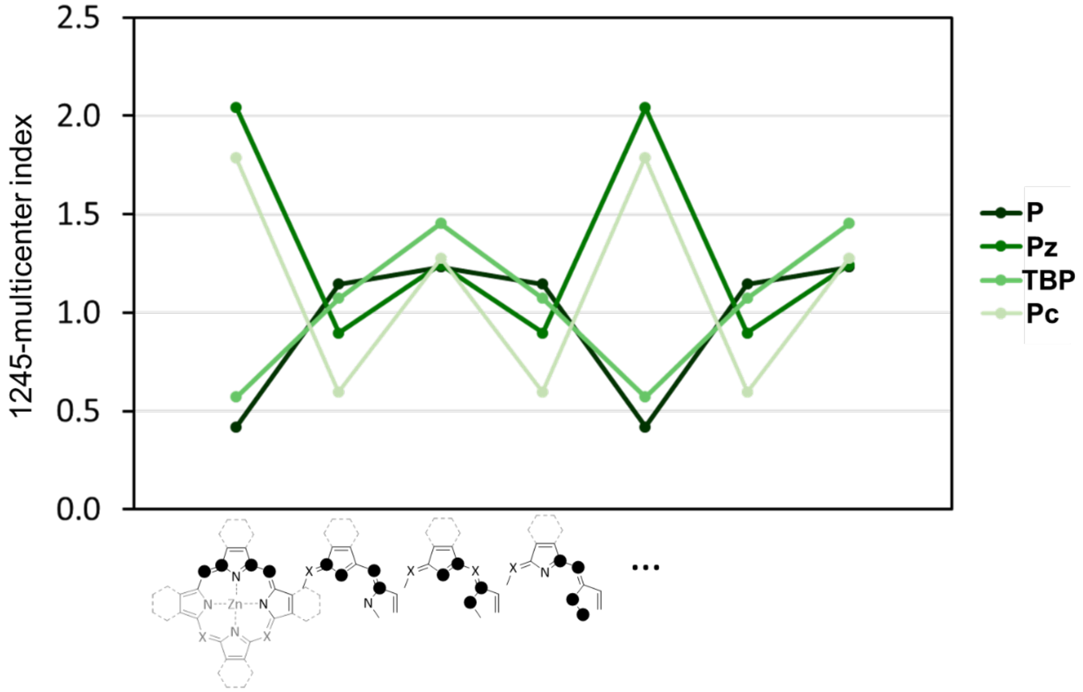

Figure S16. 1245-index distribution profile for a representative section of the iiii pathway of D_{4h} systems.

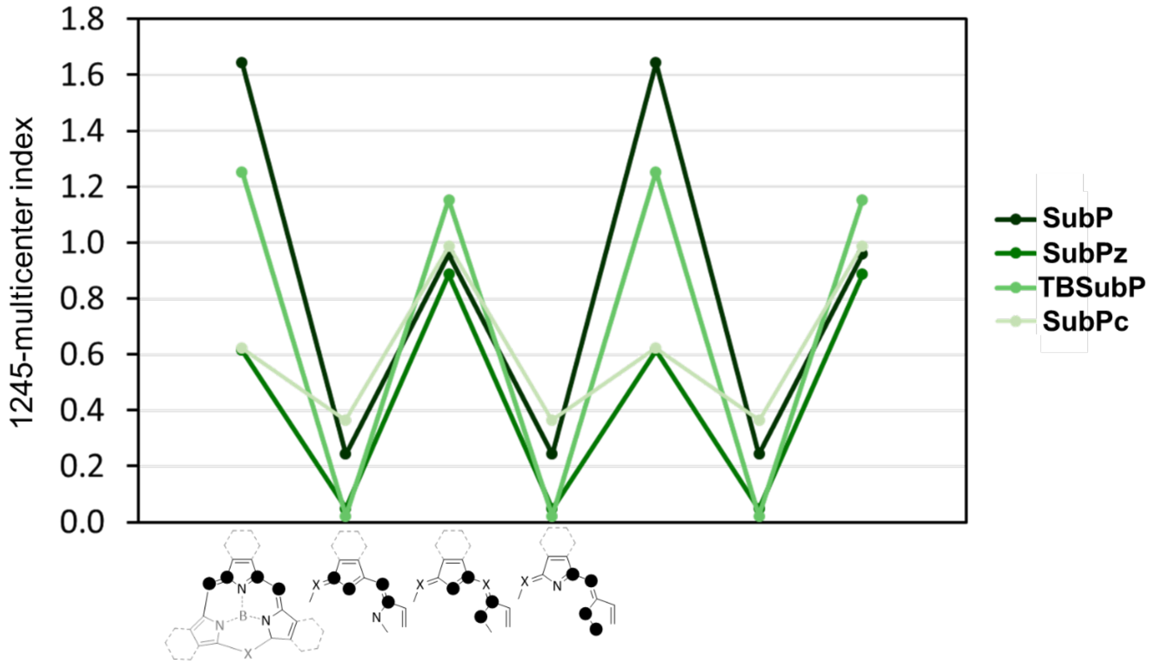

Figure S17. 1245-index distribution profile for a representative section of the iii pathway of C_{3v} systems.

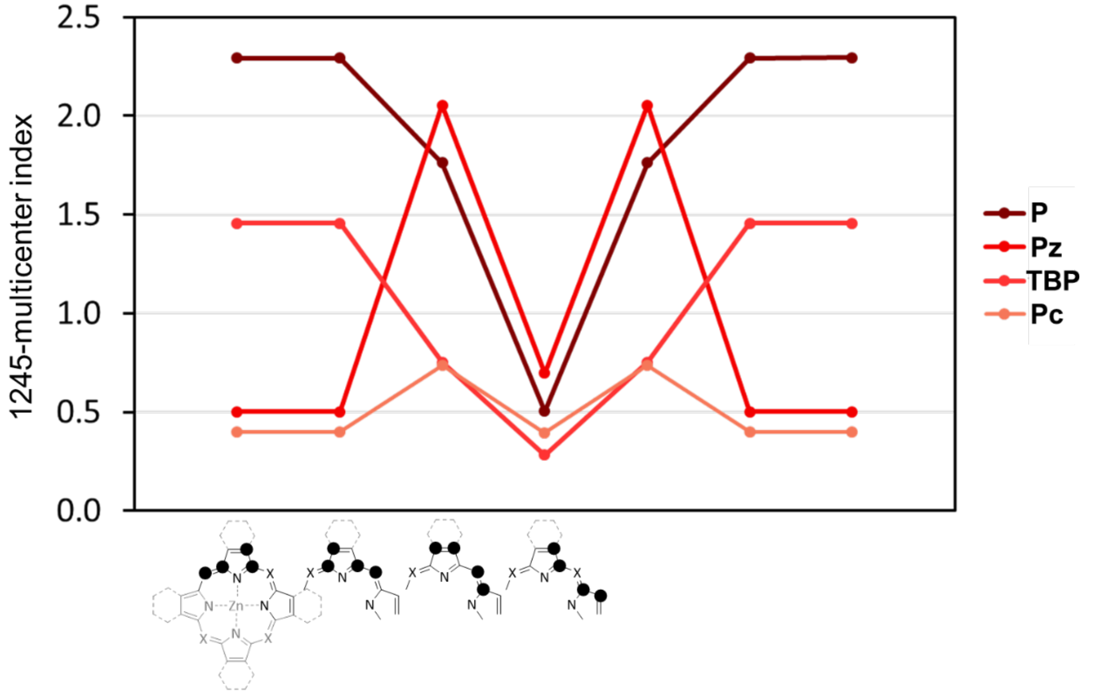

Figure S18. 1245-index distribution profile for a representative section of the oooo pathway of D_{4h} systems.

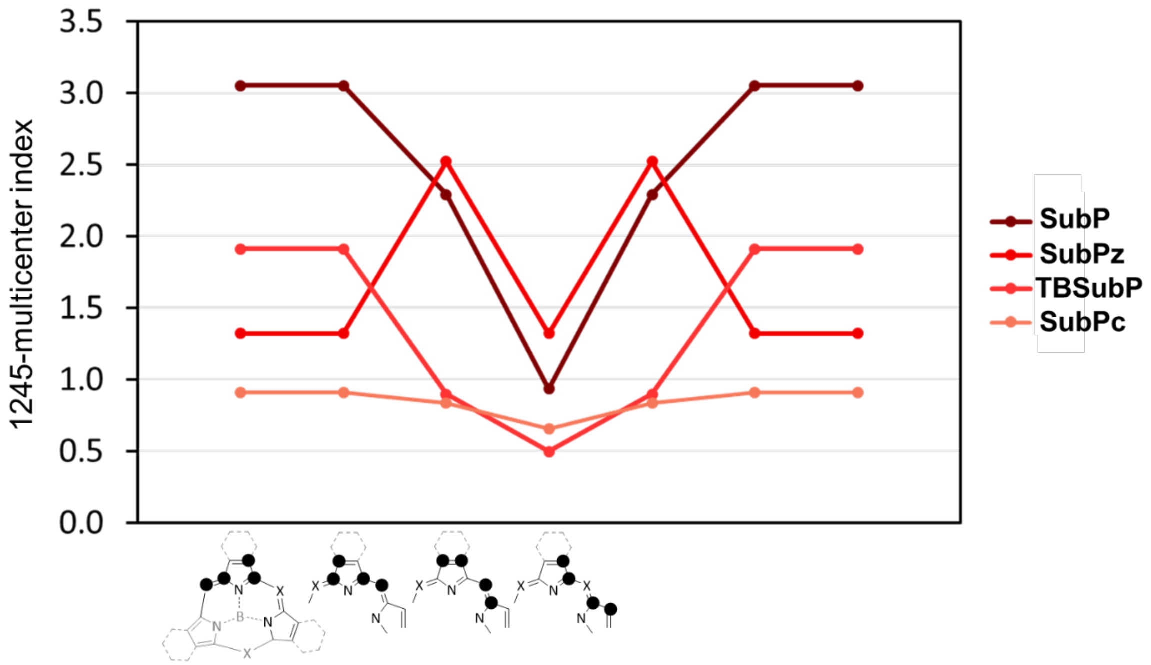

Figure S19. 1245-index distribution profile for a representative section of the ooo pathway of C_{3v} systems.

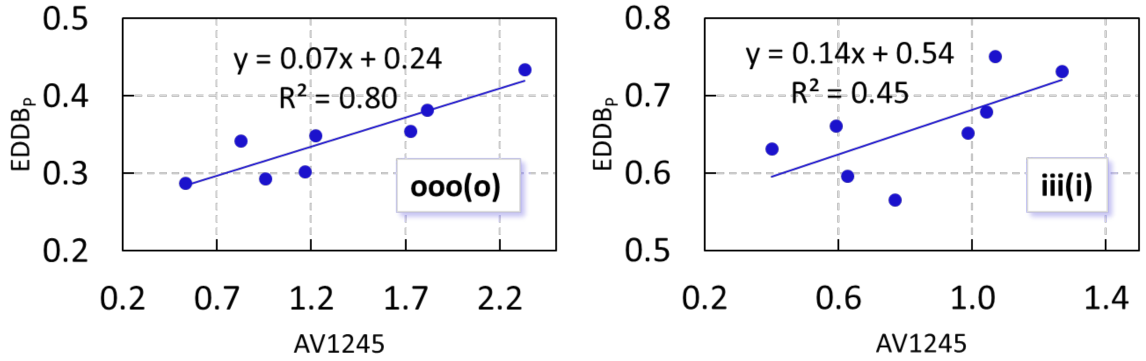

Figure S20. Linear Correlation of $EDDB_P$ and AV1245 indices for ooo(o) and iii(i) pathways.

S4.4. Solvent effects

To address the effect of axial coordination of the solvent, we performed computations in the Zn-Porphyrin system including a solvent molecule coordinated at the axial position. The summary of the results is presented in the following Figure S21.

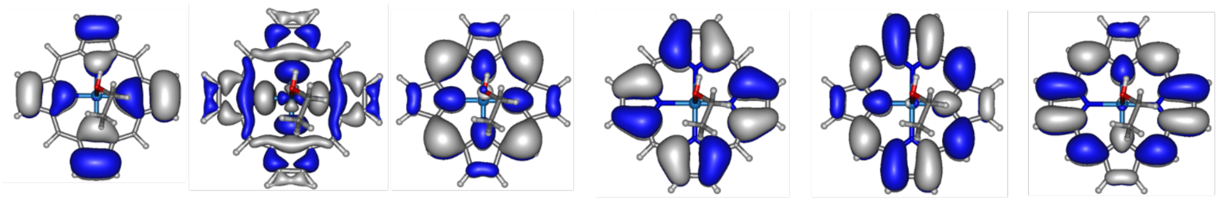

Orbital energies a.u

HOMO-3	-8.45	Occupied
HOMO-2	-8.21	Occupied
HOMO-1	-6.46	Occupied
HOMO	-6.43	Occupied
LUMO	-1.60	Unoccupied
LUMO+1	-1.59	Unoccupied

Q and B bands:

Q_1 → 2.3464 eV 528.40 nm f = 0.0059
Q_2 → 2.3516 eV 527.24 nm f = 0.0054
B_1 → 3.4500 eV 359.37 nm f = 1.2602
B_2 → 3.4500 eV 359.37 nm f = 1.2513

Figure S21. Frontier molecular orbitals, orbital energies and Q and B absorption bands for EtOH-Zn-Porphyrin.

The data show that the presence of an ethanol molecule does not affect the frontier orbitals, which remain consistent with those of the tetracoordinate system. The orbital energies are comparable to those reported in Table S17, and the Q and B bands are consistent with the tetracoordinate system as shown in Table 1 of the manuscript. Additionally, the aromaticity indices exhibit only small differences between the penta- and tetracoordinated systems, as demonstrated in Table S35.

Table S35. Differences between FLU, HOMA, AV1245 and AV_{min} aromaticity indices for tetracoordinated Zn-Phthalocianine and EtOH-Zn-Porphyrin.

Pathway	atoms	ΔFLU	ΔHOMA	ΔAV1245	ΔAV_{min}
iiii	16	-0.0002	0.007	-0.012	-0.192
oiii (4)	17	0.0004	-0.006	-0.063	-0.192
ooii (4)	18	0.0001	-0.010	-0.023	-0.065
oioi (2)	18	0.0002	-0.013	-0.051	-0.192
oooi (4)	19	0.0005	-0.019	-0.046	-0.192
oooo	20	0.0002	-0.021	-0.017	-0.056

S5. References

(1) Scheidt, W. R.; Dow, W. *J. Am. Chem. Soc.* **1977**, *99*, 1101.
(2) Kietaibl, H. *Monatsh. Chem.* **1974**, *105*, 405.
(3) Groom, C. R.; Bruno, I. J.; Lightfoot, M. P.; Ward, S. C. *Acta Crystallogr. B* **2016**, *72*, 171.
(4) Becke, A. D. *J. Chem. Phys.* **1993**, *98*, 5648.
(5) Lee, C.; Yang, W.; Parr, R. G. *Phys. Rev. B* **1988**, *37*, 785.
(6) Stephens, P. J.; Devlin, F. J.; Chabalowski, C. F.; Frisch, M. J. *J. Phys. Chem.* **1994**, *98*, 11623.
(7) Yanai, T.; Tew, D. P.; Handy, N. C. *Chem. Phys. Lett.* **2004**, *393*, 51.
(8) Chai, J.-D.; Head-Gordon, M. *J. Chem. Phys.* **2008**, *128*, 084106.
(9) Zhao, Y.; Truhlar, D. G. *Theor. Chem. Acc.* **2008**, *120*, 215.

(10) Tao, J.; Perdew, J. P.; Staroverov, V. N.; Scuseria, G. E. *Physical Review Letters* **2003**, *91*, 146401.
(11) Vydrov, O. A.; Scuseria, G. E. *J. Chem. Phys.* **2006**, *125*, 234109.
(12) Henderson, T. M.; Izmaylov, A. F.; Scalmani, G.; Scuseria, G. E. *J. Chem. Phys.* **2009**, *131*, 044108.
(13) Dunning, T. H. *J. Chem. Phys.* **1989**, *90*, 1007.
(14) Bokareva, O. S.; Grell, G.; Bokarev, S. I.; Kühn, O. *J. Chem. Theory Comput.* **2015**, *11*, 1700.
(15) Karolewski, A.; Kronik, L.; Kümmel, S. *J. Chem. Phys.* **2013**, *138*, 204115.
(16) Mack, J.; Stone, J.; Nyokong, T. *J. Porphyr. Phthalocya.* **2014**, *18*, 630.
(17) Drzewiecka-Matuszek, A.; Rutkowska-Zbik, D. *Molecules*, **2021**, *26*, 7176.
(18) Mewes, S. A.; Plasser, F.; Krylov, A.; Dreuw, A. *J. Chem. Theory Comput.* **2018**, *14*, 710.
(19) Szczepanik, D. W.; Solà, M.; Andrzejak, M.; Pawełek, B.; Dominikowska, J.; Kukułka, M.; Dyduch, K.; Krygowski, T. M.; Szatylowicz, H. *J. Comput. Chem.* **2017**, *38*, 1640.
(20) Casademont-Reig, I.; Woller, T.; Contreras-García, J.; Alonso, M.; Torrent-Sucarrat, M.; Matito, E. *Phys. Chem. Chem. Phys.* **2018**, *20*, 2787.
(21) Casademont-Reig, I.; Guerrero-Avilés, R.; Ramos-Cordoba, E.; Torrent-Sucarrat, M.; Matito, E. *Angew. Chem. Int. Ed.* **2021**, *60*, 24080.
(22) Ahrens, J. P.; Geveci, B.; Law, C. C. In *The Visualization Handbook* 2005.
(23) Kruszewski, J.; Krygowski, T. M. *Tetrahedron Lett.* **1972**, *13*, 3839.
(24) Matito, E.; Duran, M.; Solà, M. *J. Chem. Phys.* **2004**, *122*, 014109.
(25) Matito, E.; Duran, M.; Solà, M. *J. Chem. Phys.* **2006**, *125*, 059901.
(26) Matito, E. *Phys. Chem. Chem. Phys.* **2016**, *18*, 11839.
(27) García-Fernández, C.; Sierda, E.; Abadía, M.; Bugenhagen, B.; Prosenc, M. H.; Wiesendanger, R.; Bazarnik, M.; Ortega, J. E.; Brede, J.; Matito, E.; Arnau, A. *J. Phys. Chem. C* **2017**, *121*, 27118.
(28) Bultinck, P.; Ponec, R.; Van Damme, S. *J. Phys. Org. Chem.* **2005**, *18*, 706.
(29) Giambiagi, M.; de Giambiagi, M. S.; dos Santos Silva, C. D.; de Figueiredo, A. P. *Phys. Chem. Chem. Phys.* **2000**, *2*, 3381.
(30) Szczepanik, D. W.; Andrzejak, M.; Dominikowska, J.; Pawełek, B.; Krygowski, T. M.; Szatylowicz, H.; Solà, M. *Phys. Chem. Chem. Phys.* **2017**, *19*, 28970.
(31) Szczepanik, D. W.; Andrzejak, M.; Dyduch, K.; Żak, E.; Makowski, M.; Mazur, G.; Mrozek, J. *Phys. Chem. Chem. Phys.* **2014**, *16*, 20514.
(32) Szczepanik, D. W.; Żak, E.; Dyduch, K.; Mrozek, J. *Chem. Phys. Lett.* **2014**, *593*, 154.
(33) Hanwell, M. D.; Curtis, D. E.; Lonie, D. C.; Vandermeersch, T.; Zurek, E.; Hutchison, G. R. *J. Cheminform.* **2012**, *4*, 17.
(34) Martynov, A. G.; Mack, J.; May, A. K.; Nyokong, T.; Gorbunova, Y. G.; Tsivadze, A. Y. *ACS Omega* **2019**, 4, 7265.
(35) Feixas, F.; Matito, E.; Poater, J.; Solà, M. *Chem. Soc. Rev.,* **2015**, 44, 6434.
(36) Feixas, F.; Matito, E.; Poater, J.; Solà, M. *J. Comput. Chem*., **2008**, *29*, 1543.
(37) Casademont-Reig, I.; Ramos-Cordoba, E.; Torrent-Sucarrat, M.; Matito, E. Aromaticity Descriptors Based on Electron Delocalization: In Aromaticity Modern Computational Methods and Applications, pages 236–259. Elsevier, Amsterdam, **2021**.
(38) Matito, E.; Poater, J.; Duran, M.; Solà, M. *J. Mol. Struct.,* **2005**, 727, 165.
(39) Casademont-Reig, I.; Ramos-Cordoba, E.; Torrent-Sucarrat, M.; Matito, E. *Molecules*, **2020**, 25, 711.
(40) Claessens, C. G.; Hahn U.; Torres, T. *Chem. Rec*. **2008**, 8, 75.
(41) Claessens, C. G.; González-Rodríguez, D.; Rodríguez-Morgade, M. S.; Medina, A.; Torres, T. *Chem. Rev*. **2014**, 114, 2192.
